# Enhanced Antibiotics Sieving by Exfoliated TiS_2_ Membranes via Surface Functionalization and Passivation

**DOI:** 10.1007/s40820-026-02315-4

**Published:** 2026-08-14

**Authors:** Ruixin Yan, Mingzi Sun, Honglu Hu, Ruijie Yang, Zhen Zhang, Weikang Zheng, Liang Mei, Ting Ying, Yue Zhang, Alicia Kyoungjin An, Chuyang Y. Tang, Jingyun Fang, Bolong Huang, Zhiyuan Zeng

**Affiliations:** 1 Department of Materials Science and Engineering, and State Key Laboratory of Marine Environmental Health, City University of Hong Kong, 83 Tat Chee Avenue, Hong Kong, 999077 People’s Republic of China; 2 Department of Chemistry, City University of Hong Kong, Hong Kong, 999077 People’s Republic of China; 3 Department of Chemical and Biological Engineering, The Hong Kong University of Science and Technology, Hong Kong, 999077 People’s Republic of China; 4 Department of Civil Engineering, The University of Hong Kong, Hong Kong, 999077 People’s Republic of China; 5 Guangdong Provincial Key Laboratory of Environmental Pollution Control and Remediation Technology, School of Environmental Science and Engineering, Sun Yat-Sen University, Guangzhou, 510275 People’s Republic of China; 6 Shenzhen Research Institute, City University of Hong Kong, Shenzhen, 518057 People’s Republic of China

**Keywords:** TiS_2_ membranes, Antibiotic removal, Antioxidation passivation, Molecular sieving

## Abstract

Transition metal dichalcogenide (TMD) nanolaminate membranes hold great promise for molecular sieving due to their two-dimensional capillary structures, which facilitate size-restricted diffusion. However, many transition metal sulfides exhibit intrinsic instability and are highly susceptible to oxidation, which severely limits their durability under reverse osmosis operating conditions. In this work, we introduce a stable 1T phase titanium disulfide (TiS_2_) constructing nanolaminate membranes from Group IVB, enabling the formation of tunable capillary channels and achieving a permeance up to 46.8 L m^−2^ h^−1^ bar^−1^. Experiments confirm that water-initiated hydrolysis, rather than direct oxidation by molecular O_2_, dominates the degradation of TiS_2_, while alkyl-chain hydrophobic barriers effectively suppress this water-triggered degradation. The enhanced surface hydrophobic properties simultaneously improve antioxidation stability and ensure sustained performance over 30 filtration cycles and 15 days of continuous operation. Functionalized membranes further reached 96.9% rejection for diverse antibiotics, such as sulfamethoxazole, ciprofloxacin, tetracycline, and rifampicin. This work opens new avenues for exploring emerging TMD materials in the efficient and reliable removal of trace antibiotics from water, contributing to safer water resources and promoting sustainable ecological practices.

**Supplementary Information:**

The online version contains supplementary material available at 10.1007/s40820-026-02315-4.

## Highlights

- A facile electrostatic self-assembly strategy simultaneously achieves interlayer expansion and antioxidation passivation of TiS_2_ membranes via quaternary ammonium functionalization.
- The optimized DD-TiS_2_ membrane exhibits > 95% antibiotic rejection with high water permeance and retains stable performance over 30 filtration cycles and 15 days of continuous operation.
- Density functional theory and molecular dynamics simulations reveal that hydrophobic alkyl-chain barriers suppress water-initiated TiS_2_ hydrolysis, while tunable capillary width governs antibiotic rejection via geometric size sieving.

**Supplementary Information:**

The online version contains supplementary material available at 10.1007/s40820-026-02315-4.

## Graphical abstract

**Figure:**
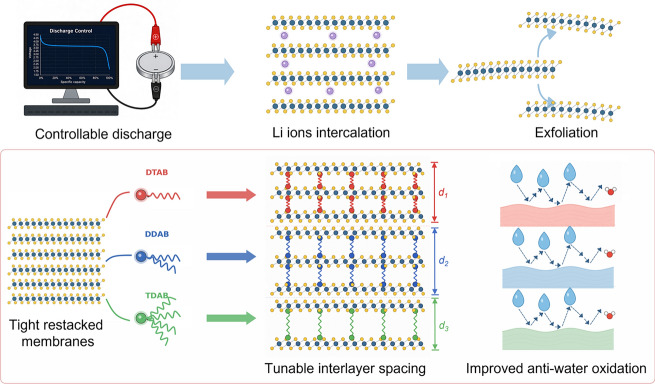

## Introduction

Pharmaceutical contamination in water poses a growing threat to public health and ecosystems, with small-molecule antibiotics, being especially difficult to remove because they readily permeate conventional treatment barriers and can foster the spread of antibiotic-resistant bacteria [1–3]. Although biological treatment, adsorption, and advanced oxidation processes have been widely explored for antibiotic removal, each suffers from intrinsic limitations in selectivity, energy demand, or secondary waste generation [4–7]. Reverse osmosis (RO) membrane separation, by contrast, offers the most promising combination of broad molecular applicability and high rejection efficiency for trace pharmaceuticals [8, 9]. Yet the performance of RO membranes is ultimately governed by the durability of the active layer under prolonged high-pressure aqueous operation—particularly its resistance to oxidation in oxygen- and water-rich environments—which remains the critical bottleneck for the practical deployment of next-generation membrane materials.

Among emerging two-dimensional and porous membrane platforms—including metal–organic and covalent organic frameworks [10, 11], layered double hydroxides [12], reduced graphene oxide [5, 13, 14], and novel polymer composites [15–19]—transition metal dichalcogenide (TMD) nanolaminates have attracted particular interest for molecular sieving owing to their well-defined two-dimensional capillary channels. Recent studies regarding graphene-based and carbon-based nanomaterials further show how surface chemistry and nanoscale patterning regulate antibacterial activity, bio-interface assembly, and catalytic redox functionality, providing useful context for designing chemically stable 2D interfaces [20–24]. However, the most widely studied TMDs, MoS_2_ and WS_2_ [25–29], suffer from an intrinsic crystallographic instability during lithium intercalation-based exfoliation [30–34]: The parent 2H phase is converted to a metastable 1T′ phase that progressively reverts and degrades under aqueous operation, ultimately compromising both structural integrity and long-term separation performance [35, 36]. In contrast, Group IVB TMD such as TiS_2_ retains a thermodynamically stable 1T phase throughout exfoliation and post-modification [37], providing a more robust crystallographic foundation for RO membrane engineering. The remaining challenge for TiS_2_ is its inherent water sensitivity: Surface hydroxylation drives conversion to TiO_2_ with concurrent H_2_S release [38, 39], disrupting the layered architecture and limiting long-term durability. Establishing an effective antioxidation strategy is therefore essential for translating TiS_2_-based nanolaminate membranes into practical RO platforms.

Compared with prior MoS_2_-based membrane works that primarily relied on covalent functionalization to tune interlayer spacing or on defect passivation to improve stability as separate objectives, our approach achieves both simultaneously through a single electrostatic self-assembly step [40]. Moreover, TiS_2_ faces a distinct degradation pathway—hydroxylation-driven conversion to TiO_2_ with H_2_S release—that is mechanistically different from the oxidation pathways of MoS_2_, and our passivation strategy is specifically designed to address this water sensitivity challenge [38].

The selection of Group IVB TiS_2_ is further supported by its thermodynamic preference for the 1T polytype: As a d^0^ transition metal, Ti^4+^ favors octahedral coordination with sulfur ligands according to crystal field theory, whereas d^2^ metals such as Mo^4+^ prefer the trigonal prismatic 2H arrangement [41]. Consequently, 1T-TiS_2_ is the thermodynamically stable bulk polytype under ambient conditions, and the 1T phase is preserved throughout electrochemical lithium intercalation–exfoliation, in agreement with previous reports on Group IVB TMDs [42]. This intrinsic 1T phase stability—a distinguishing feature of Group IVB TMDs relative to the metastable 1T’ phases of MoS_2_/WS_2_ obtained by intercalation—provides the crystallographic foundation on which our post-filtration passivation strategy operates [43].

In this work, we developed a post-passivation strategy using quaternary ammonium molecules including dodecyltrimethylammonium bromide (DTAB), didodecyldimethylammonium bromide (DDAB), and tetra-n-dodecylammonium bromide (TDAB). The selection of these three modifiers was designed to systematically vary the spatial configuration around the quaternary ammonium headgroup while keeping the alkyl-chain length (C_12_) constant: DTAB has one long chain, DDAB has two, and TDAB has four. This enables isolation of the effect of headgroup bulkiness on interlayer expansion and surface hydrophobicity, establishing a clear structure–property relationship between molecular architecture and membrane performance. This strategy stabilizes TiS_2_ nanosheets against water-induced oxidation, retaining ~ 87% of TiS_2_ in the unoxidized state. In addition, the capillary widths were significantly increased by expanding the capillary width from ~ 0.02 nm to up to 1.09 nm, which is a critical factor governing molecular transport. The fabricated membranes achieved efficient removal of various sizes of antibiotics including sulfamethoxazole (SMX), ciprofloxacin (CIP), tetracycline (TC), and rifampicin (RIF) with superior stability. Mechanistically, this antioxidation enhancement is rationalized through a combined experimental and computational framework: Time-resolved UV–vis spectroscopy and X-ray photoelectron spectroscopy (XPS) track the surface oxidation kinetics, humidity-controlled exposure tests decouple the contributions of molecular O_2_ and water vapor and identify water-initiated hydrolysis as the dominant degradation pathway, and density functional theory (DFT) together with molecular dynamics (MD) simulations quantify the suppressed water adsorption energy and elevated diffusion barrier on the alkyl-passivated surface. This integrated approach establishes a generalizable post-filtration passivation protocol for water-sensitive TMD nanolaminate membranes and clarifies the chemical origin of their long-term operational stability.

## Experimental Section

### Materials

All chemicals from chemical suppliers were used without further purification. Titanium disulfide (TiS_2_) bulk powder was purchased from Sigma-Aldrich. Rifampicin, tetracycline hydrochloride, ciprofloxacin, sulfamethoxazole, dodecyltrimethylammonium bromide, didodecyldimethylammonium bromide, tetra-n-dodecylammonium bromide were purchased from Macklin. Hydrochloric acid (HCl) solution (35–38 wt%), sodium hydroxide (NaOH), polyvinylidene fluoride (PVDF) were purchased from Aladdin.

### Preparation of TiS_2_ Nanosheets and Membranes

The TiS_2_ nanosheets were prepared via previous reported electrochemical lithium intercalation and exfoliation methods. Briefly, a slurry consisting of 80 wt.% bulk TiS_2_ powder, 10 wt.% super P (Imerys, France), and 10 wt.% polyvinylidene difluorides (PVDF (Sigma, China)) was mixed in a research bowl, followed by the addition of an appropriate amount of NMP for auxiliary dissolution. Finally, it is uniformly coated on aluminum foil and vacuum-dried at 100 degrees for 12 h. The CR2025 cells (Guangdong Canrd New Energy Technology Co. Ltd., China) were assembled in a glove box filled with argon gas, using TiS_2_ electrode as the working electrode, lithium foil (China Energy Lithium Co., Ltd) as the counterelectrode as well as reference electrode, and 1 M LiPF_6_ in ethylene carbonate (EC): ethyl methyl carbonate (EMC): dimethyl carbonate (DMC) (1:1:1 in volume) as electrolyte (Suchow DoDoChem Technology) and PP film (Celgard 2300) as the separator. The galvanostatic discharge was conducted using the Neware battery testing system (Neware, China) at room temperature. The current was 0.1C and the cutoff voltage was 0.9 V. The TiS_2_ nanosheets were acquired by sonication of the lithiated sample (Li_*x*_TiS_2_) in deionized water. The produced gas (H_2_) aids in the exfoliation of bulk TiS_2_ into individual monolayer nanosheets. Subsequently, the suspension of these TiS_2_ nanosheets was purified through centrifugation. A TiS_2_ membrane was then created by vacuum filtration of the refined TiS_2_ nanosheets solution onto a PVDF substrate (220 nm pore size, 25 mm in diameter). The thickness was adjusted by regulating the volume of the filtered solution.

### Preparation of Functionalized TiS_2_ Membranes

An ethanol solution containing an equal amount of 0.1 mM DTAB/DDAB/TDAB was mixed with 2 mL of water and subjected to vacuum filtration on the surface of a freshly wet TiS_2_ membrane. Following filtration, an additional 20 mL of water was used for rinsing to remove any unreacted modifiers. Subsequently, the modified membrane was subjected to vacuum drying at 100 °C for 12 h in a vacuum oven.

### Characterizations

XPS was conducted using Thermo Fisher Scientific K-Alpha + (Thermo Fisher Scientific, USA) with Al Kα (hυ = 1486.6 eV) X-ray source. Raman spectra of TiS_2_ membrane were collected at room temperature using a Renishaw (Renishaw, UK) via Raman microscope with a × 50 objective lens and lateral spatial resolution of around 1 μm. XRD data were collected using a Bruker D8 diffractometer with Cu^−^Kα radiation (Bruker, USA). SEM images were obtained using a Scanning Electron Microscope (SEM) (TESCAN MIRA). The transmission electron microscopy (TEM) images were taken on transmission electron microscopes (FEI, Talos). UV–Vis absorption test was taken on Hitachi UH4150 UV–VIS-NIR Spectrophotometer. Total organic carbon test was taken on Shimadu TOC-L CPH.

### Antioxidant Performance Tests

Two bottles of pure TiS_2_ ink solution were diluted in equal proportions, with DDAB subsequently added to one bottle, while the other bottle served as a blank control group. The change in the UV absorption peak intensity at 580 nm over time was then compared to assess the extent of the TiS_2_ -water reaction. The antioxidant testing of the modified membrane included immersing the TiS_2_ membrane in pure water for 48 h and observing any changes in color and structure on the membrane surface. The reverse osmosis (RO) antioxidant testing involved continuous filtration of ultrapure water through the TiS_2_ membrane for 24 h, with a comparison made between the color and structure changes in the area in contact with water.

### Calculation Setup

To investigate the influences of surface modifications induced by DTAB, DDAB, and TDAB, we performed density functional theory (DFT) calculations using the CASTEP package to better understand the reaction mechanism. We have applied the generalized gradient approximation (GGA) and Perdew–Burke–Ernzerhof (PBE) functionals to properly describe the exchange–correlation interactions. For the geometry optimizations, the cutoff energy has been set to 380 eV based on the ultrafine quality and the ultrasoft pseudopotentials. The k-point is applied with coarse quality based on the Broyden–Fletcher–Goldfarb–Shannon (BFGS) algorithm to realize efficient energy minimizations. We have applied the following convergence criteria for all the geometry optimizations, where the Hellmann–Feynman forces should not exceed 0.001 eV Å^−1^, and the total energy difference should be smaller than 5 × 10^–5^ eV atom^−1^. For the practical ion strength and pH values, the corresponding calculation loading will be ultrahigh to reproduce the exact pH environment and ion concentration through the explicit model, which are also very difficult for current DFT calculations. Although there are some factors that have not been included, our current simulations still capture the interfacial water structuring, relative hydration interaction strength, and comparative energy barriers for water diffusion.

For interactions between water droplets and TiS_2_/modified TiS_2_, we have applied molecular dynamics (MD) simulations. The initial water droplets are constructed with 142 water molecules. The MD simulations are performed based on the NVT mode at 298 K for 200 ps. The simulation step is 1 fs, and the total simulation time is 200 ps with 200,000 steps. The near-surface water concentrations are calculated based on the number of water molecules located within the range of 10 Å from the surface S sites of the TiS_2_. For the interactions of TiS_2_ and TiS_2_-DDAB in the water solution by MD simulations, we have built a 40 × 40 × 40 Å^3^ box with 1607 water molecules with random distributions. The MD simulations are performed based on the NVT mode at 323 K. The simulation step is 1 fs, and the total simulation time is 10 ps with 10,000 steps. The Nose mode is applied for the temperature control during all the MD simulations. The calculation of interaction energies is listed as follows:

1 $${E}_{\mathrm{i}\mathrm{n}\mathrm{t}\mathrm{e}\mathrm{r}\mathrm{a}\mathrm{c}\mathrm{t}\mathrm{i}\mathrm{o}\mathrm{n}}={E}_{\mathrm{t}\mathrm{o}\mathrm{t}\mathrm{a}\mathrm{l}}-({E}_{Ti{S}_{2}/DD-Ti{S}_{2}}+{E}_{\mathrm{W}\mathrm{a}\mathrm{t}\mathrm{e}\mathrm{r}})$$

where ${E}_{\mathrm{i}\mathrm{n}\mathrm{t}\mathrm{e}\mathrm{r}\mathrm{a}\mathrm{c}\mathrm{t}\mathrm{i}\mathrm{o}\mathrm{n}}$ represents the energy of the interaction, ${E}_{\mathrm{t}\mathrm{o}\mathrm{t}\mathrm{a}\mathrm{l}}$ represents the total energy of the water, and DD-TiS_2_/TiS_2_, ${E}_{{\mathrm{TiS}}_{2}/\mathrm{D}\mathrm{D}-{\mathrm{TiS}}_{2}}$ represents the energy of the TiS_2_ and DD-TiS_2_, and ${E}_{\mathrm{W}\mathrm{a}\mathrm{t}\mathrm{e}\mathrm{r}}$ represents the energy of all water molecules.

### Techno-Economic Assessment Setup

To evaluate the economic feasibility of functionalized TiS_2_ membranes for antibiotic separation, a comprehensive techno-economic assessment (TEA) was performed based on the annuity techno-economic assessment method. This methodology allows for the estimation of total production costs by considering the amortization of capital investments and ongoing operational expenditures over the project’s lifecycle.

#### Analysis Indicators and Formulas

The annual capital investment cost is calculated assuming funds are borrowed at a fixed interest rate and repaid over the factory's operational lifespan. The Annualized Capital Cost (ACC) is determined as follows:

2 $$\mathrm{A}\mathrm{C}\mathrm{C}=\mathrm{F}\mathrm{C}\mathrm{I}\times \frac{\mathrm{N}\mathrm{I}\mathrm{R}\times (1+\mathrm{N}\mathrm{I}\mathrm{R}{)}^{N}}{(1+\mathrm{N}\mathrm{I}\mathrm{R}{)}^{N}-1}$$

where FCI: Fixed Capital Investment. NIR: Nominal Interest Rate. *N*: Project lifetime or plant lifecycle (years).

The Total Unit Production Cost (*C*_total_) is then calculated by summing the annualized capital costs and the cash cost of operating, divided by the total annual output:

3 $${C}_{\mathrm{t}\mathrm{o}\mathrm{t}\mathrm{a}\mathrm{l}}=\frac{(\mathrm{A}\mathrm{C}\mathrm{C}+\mathrm{C}\mathrm{C}\mathrm{O}\mathrm{P})}{\mathrm{A}\mathrm{n}\mathrm{n}\mathrm{u}\mathrm{a}\mathrm{l}\,\mathrm{P}\mathrm{r}\mathrm{o}\mathrm{d}\mathrm{u}\mathrm{c}\mathrm{t}\mathrm{i}\mathrm{o}\mathrm{n}}$$

where CCOP represents the Cash Cost of Operating, including maintenance, utilities, labor, and management fees.

#### Assessment Procedure

The cost–benefit analysis followed a standardized three-step procedure

1. Objective and Scope Definition: Clearly identifying the primary goals and boundaries of the antibiotic separation process.
2. Identification of Costs and Benefits: Collecting comprehensive data on investment costs (e.g., equipment procurement, construction) and operating costs (e.g., maintenance and management fees). The analysis considers the full lifecycle, from the initial investment stage to operation and eventual disposal.
3. Present Value Conversion: To account for the time value of money, future costs and benefits are discounted to their current value, ensuring a rigorous comparison between total expenditures and economic returns.

### Antibiotics Desalination Test

A modified TiS_2_ membrane, with an effective diameter of 1 cm, was affixed to a dead-end device, positioning the permeate side under a high pressure approximately 6 bar to assess its separation performance and stability. For the stability test, a 50 mL feed solution containing TC was employed for each cycle, containing antibiotics at a concentration of 25 ppm unless specified otherwise. Following a single cycle, the TiS_2_ membrane underwent a blank RO with 50 mL DI water before undergoing another filtration cycle. The permeance (P, in units of L m^−2^ h^−1^ bar^−1^) was determined using the equation provided below:

4 $$P = V/\left( {A \times t \times \Delta P} \right)$$

V represents the volume in the permeate side (in liters), A stands for the effective surface area of the membrane (in square meters), t denotes the duration of filtration (in hours), and ∆P signifies the applied transmembrane pressure (in bars). Antibiotic rejection is calculated using Eq. (2) as outlined below:

5 $$R = (1 - \, C_{{\mathrm{p}}} /C_{{\mathrm{f}}} ) \times 100\%$$

where *C*_f_ and *C*_p_ are the antibiotic concentration in the feed side and permeate side measured by UV–Vis spectroscopy, respectively.

## Results and Discussion

### Characterization of Restacked TiS_2_ Membrane Structure

**Fig. 1:**
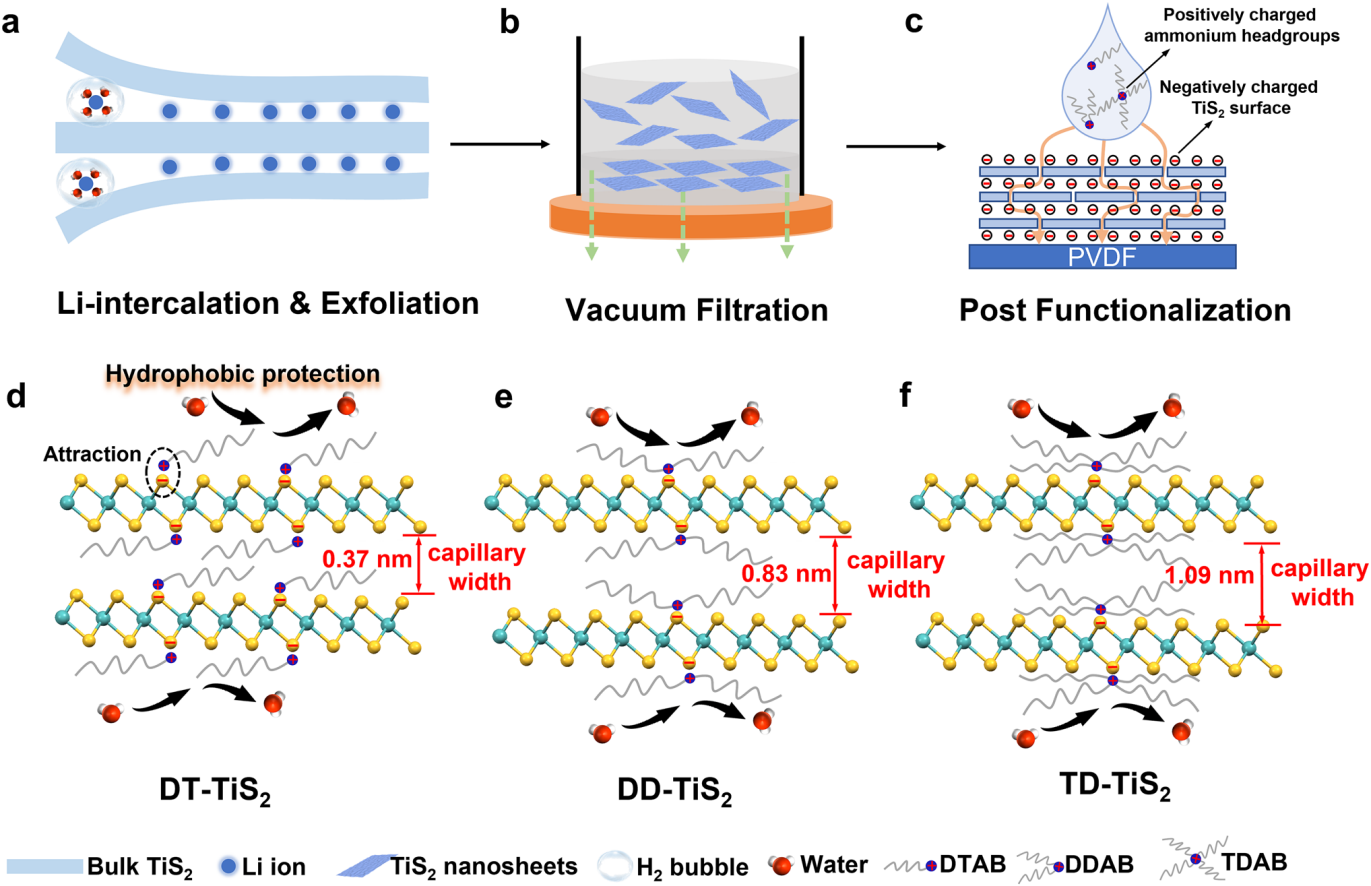
Schematic illustration of interlayer spacing regulation and water transport in functionalized TiS_2_ membranes. **a** Li-ion intercalation and exfoliation of bulk TiS_2_ powders. **b** Vacuum filtration of retacking TiS_2_ membrane. **c** Post-functionalization of TiS_2_ membrane by filtration quaternary ammonium solution. Schematics of dual function of hydrophobic protection and interlayer spacing expansion by **d** DTAB, **e** DDAB, **f** TDAB molecules. In the legend, each quaternary ammonium modifier is depicted as an individual molecule with its cationic N^+^ headgroup electrostatically anchored to the negatively charged TiS_2_ surface and the hydrophobic alkyl tails oriented outward; these symbols denote individually adsorbed molecules rather than pre-formed micellar aggregates. The assembly proceeds by electrostatic adsorption followed by spontaneous self-assembly of the alkyl chains, consistent with the main-text description

**Fig. 2:**
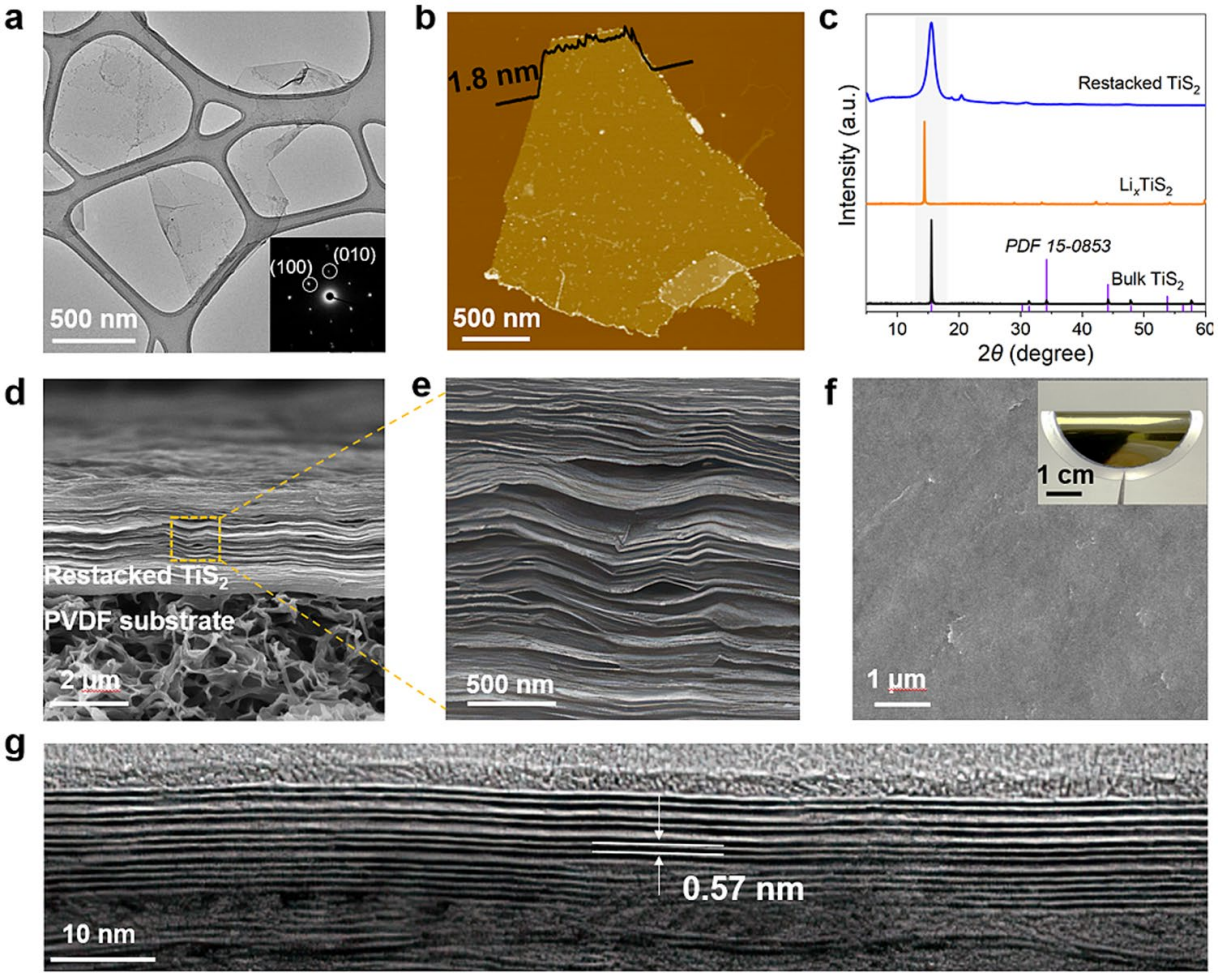
Characterizations of TiS_2_ nanosheets and unmodified membranes. **a** TEM image of TiS_2_ nanosheet and its corresponding SAED pattern. **b** AFM image of pristine TiS_2_ nanosheet. **c** XRD patterns of bulk TiS_2_ powder, its Li-intercalated Li_*x*_TiS_2_ and restacked TiS_2_ membrane. **d, e** Cross-sectional SEM image of TiS_2_ membrane and its magnified image. **f** Top-view SEM image of TiS_2_ membrane, inset is its digital photograph. **g** Cross-sectional HRTEM image of the TiS_2_ membrane

High-quality few-layer TiS_2_ nanosheets were obtained from their bulk counterparts (Fig. S1) by an electrochemical lithium-ion (Li^+^) intercalation-based exfoliation method, in which a galvanostatic discharge was applied with a cutoff voltage of 0.9 V [41, 44–46] (see Methods for details). Subsequently, the Li-intercalated Li_*x*_TiS_2_ electrodes were soaked in DI water, where the formation of hydrogen bubbles together with ultrasonic agitation facilitated the disruption of the van der Waals forces between the layers (Fig. 1a). A nanolaminate membrane with an ordered nanoscale layered architecture was fabricated via a vacuum filtration process, which aligned the randomly restacked nanosheets into a well-organized laminar structure (Fig. 1b). Then, ethanol–water mixtures containing different quaternary ammonium salt molecules (DTAB, DDAB, and TDAB) were used for post-filtration modification (Fig. 1c). The post-filtration approach was specifically adopted over pre-mixing or co-assembly methods to preserve membrane integrity: Introducing quaternary ammonium molecules prior to filtration would drastically shift the zeta potential from negative to positive, causing premature nanosheet aggregation and disordered stacking. The passivation of the negatively charged nanosheets occurred rapidly through electrostatic adsorption of the positively charged quaternary ammonium termini, leading to the spontaneous self-assembly behavior of the hydrophobic alkyl chains on the nanosheet surface. Here, the three quaternary ammonium molecules possess distinct long-chain alkyl configurations (Fig. S2), thereby enabling systematic control over their spatial dimensions. Here, the interlayer spacing (*d*) refers to the center-to-center distance between adjacent TiS_2_ layers as measured by XRD or HRTEM, and the capillary width (*δ*) is defined as the effective free space for molecular transport, calculated as *δ* = *d* − *t*, where *t* is the thickness of a single TiS_2_ monolayer (~ 0.57 nm for 1T-TiS_2_). Based on the interlayer spacing obtained by XRD and TEM characterization, the capillary width of the functionalized TiS_2_ increased markedly from 0.02 nm (pristine restacked TiS_2_) to 0.37 nm (DT-TiS_2_), 0.83 nm (DD-TiS_2_), and 1.09 nm (TD-TiS_2_), respectively (Fig. 1d–f). Prior to modification, exfoliated pristine mono- or few-layered TiS_2_ nanosheets were also characterized by transmission electron microscopy (TEM, Figs. 2a and S3a) and atomic force microscopy (AFM, Fig. 2b). For comparison, the lithium-ion intercalation expanded the interlayer spacing with the (001) plane shifting from 15.5° to 14.4°, as confirmed by X-ray diffraction (XRD) data, and the TiS_2_ (001) peak returned to its original position upon restacking the exfoliated TiS_2_ nanosheets (Fig. 2c). The 1T phase TiS_2_ bulk and exfoliated nanosheets exhibited the same Raman vibration peak (Fig. S3b), and X-ray photoelectron spectroscopy (XPS) (Fig. S4) revealed a certain degree of oxidation during synthesis, as the characteristic peak of TiO_2_ appeared in XPS spectra. The restacked pure TiS_2_ membrane showed a distinct layered structure with no apparent particle impurities as evidenced by cross-sectional scanning electron microscopy (SEM) images (Figs. 2d, e and S5); the top-view SEM image and photograph of the membrane revealed a flat surface with a pronounced metallic luster and good flexibility (Fig. 2f). The interlayer spacing of the pristine TiS_2_ was measured to be 0.57 nm (Fig. 2g).

### Enhanced Antioxidation Properties of TiS_2_

**Fig. 3:**
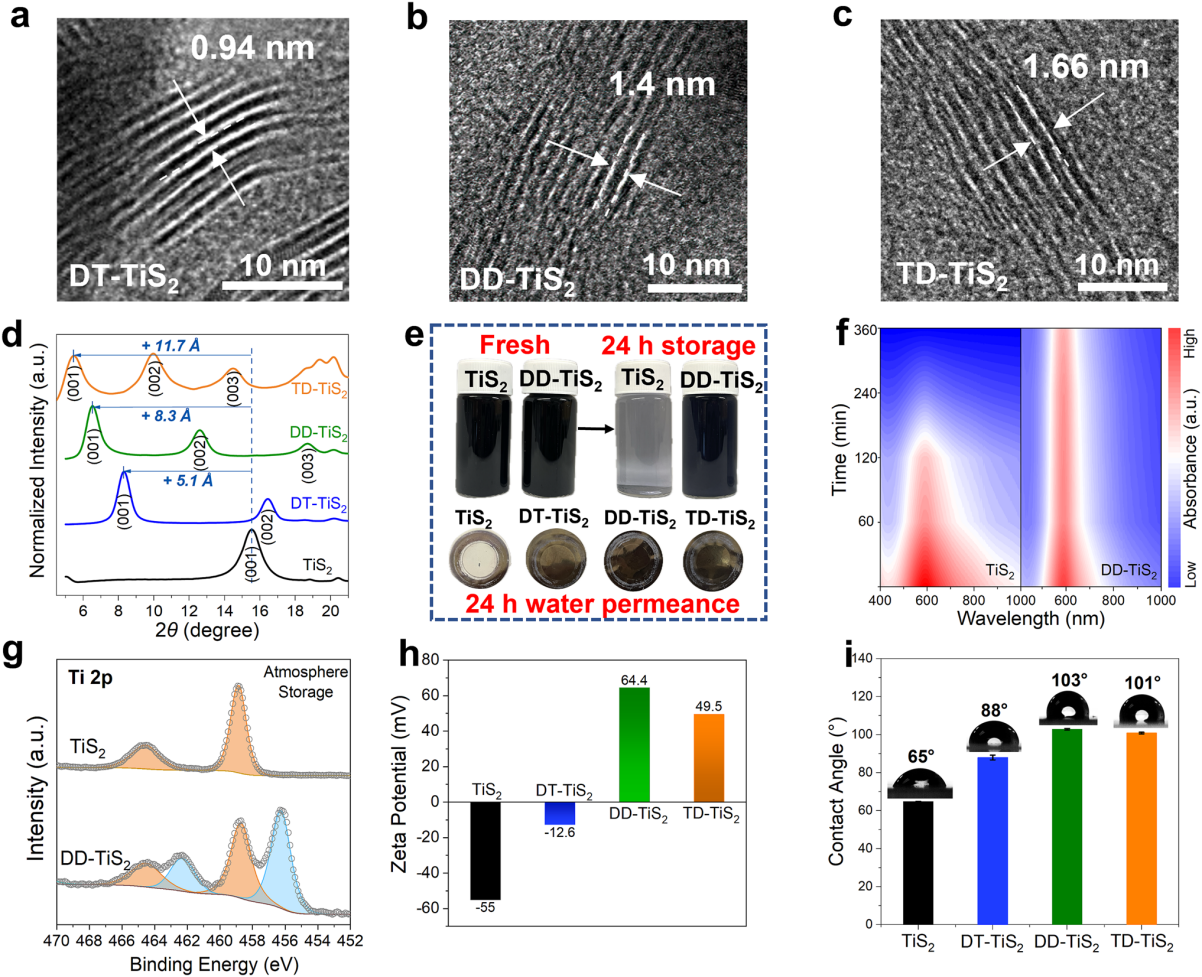
Characterizations of quaternary ammonia stabilized TiS_2_ membranes. **a-c** HRTEM image of DTAB, DDAB, TDAB decorated TiS_2_ membranes and its corresponding cross-sectional interlayer space. **d** XRD patterns of functionalized TiS_2_ membranes. **e** images of pristine TiS_2_ nanosheets ink solution and DD-TiS_2_ solution after 24 h water storage, pristine TiS_2_ and three functionalized membranes after 24-h RO test (the central circle was the water permeance area). Quaternary ammonium functionalization prevents water-induced oxidation of TiS_2_ observed by unchanged color. **f** In-situ UV–vis absorbance of pristine TiS_2_ nanosheets and DD-TiS_2_ nanosheets in water. **g** XPS Ti 2*p* spectra of pristine and DD-TiS_2_ storage in atmosphere for 24 h. **h** Zeta potential of pristine and functionalized TiS_2_ nanosheets (0.1 mg mL^−1^ in DI water, natural pH ~ 6.5, no background electrolyte, 25 °C). **i** surface contact angle of pristine and functionalized TiS_2_ membranes

XPS analysis confirmed the successful functionalization of quaternary ammonium molecules on the TiS_2_ nanosheet surface through the detection of a nitrogen signal (Fig. S6a-c). The formation of the hydrophobic protective layer was evidenced by the uniformly distributed nitrogen element in Energy-dispersive X-ray spectroscopy (EDS) mapping of the TiS_2_ nanosheets (Fig. S6d). The DT-TiS_2_, DD-TiS_2_, and TD-TiS_2_ membranes all exhibited a well-restacked layered structure in cross-sectional views, with expanded interlayer spacing of 0.94, 1.4, and 1.66 nm, respectively (Fig. 3a–c). This expansion of interlayer spacing was further confirmed by XRD data where the (001) plane that shifted systematically to lower diffraction angles for functionalized TiS_2_ membranes (Fig. 3d). All the functionalized membranes exhibited gold-like metallic luster due to their semi metallic property along with high flexibility as shown in Fig. S7. The unchanged Raman peaks before and after exfoliation of the TiS_2_ indicated that the exfoliation process did not alter its intrinsic structural or chemical characteristics (Fig. S8). Furthermore, Fourier transform infrared spectroscopy (FTIR) revealed enhanced methylene peaks, confirming the successful grafting of alkyl chains between the membrane layers (Fig. S9). Notably, after DDAB modification, the TiS_2_ membrane thickness increased from 2.2 to 4.6 µm, further demonstrating effective intercalation and structural expansion induced by DDAB functionalization (Fig. S10).

In contrast, as shown in Fig. 3e, after 24 h dispersion in water, the pristine TiS_2_ colloid faded to a transparent state due to its oxidation to TiO_2_. The intrinsic instability of unmodified TiS_2_ is clearly manifested by its morphological degradation after water exposure. As shown in Fig. S11a–d, pristine TiS_2_ nanosheets exhibit a smooth surface in AFM, a defect-free lattice in TEM, and a well-aligned lamellar stacking structure in cross-sectional SEM, accompanied by a sharp (001) diffraction peak in XRD. These results confirm the highly ordered layered architecture of the fresh TiS_2_ membrane. In contrast, pronounced structural degradation was observed after oxidation and RO operation (Fig. S11e–h). AFM images reveal widespread surface pitting and nanoscale voids, indicating oxidation-induced etching and nonuniform surface. TEM further shows the emergence of voids and contrast inhomogeneity, reflecting disruption of lattice continuity and local structural damage. Consistently, cross-sectional SEM images display a disordered lamellar structure with collapsed galleries and interlayer debris, while the weakened (001) diffraction indicates a gradual loss of layered ordering upon oxidation. In contrast, DD-TiS_2_ retained its original dark blue color. In the bottom side image of Fig. 3e, after a 12 h’ RO test, the permeance area of the pristine TiS_2_ membrane had already changed to a white color (TiO_2_), while functionalized TiS_2_ membranes preserved the original golden color without decay. The improved antioxidation performance of TiS_2_ nanosheets modified with quaternary ammonium molecules was further evidenced by the lack of visible oxidation pores on their surface (Fig. S12a-c). Additionally, AFM images clearly show that all three ammonium-functionalized TiS_2_ nanosheets possessed smooth and uniform surfaces, accompanied by an increase in overall thickness of ~ 3 nm (DT-TiS_2_), ~ 4 nm (DD-TiS_2_), and ~ 6 nm (TD-TiS_2_). This thickness increment directly reflected the formation of an organic protection layer on the TiS_2_ surface, originating from the grafted ammonium modifiers. Such a layer serves to passivate surface defects, enhance environmental stability, and prevent oxidation of the ultrathin TiS_2_ sheets (Fig. S12d-i). Furthermore, ultraviolet (UV) absorption was used to evaluate the stability of the TiS_2_ nanosheets dispersion, since the absorbance intensity showed a strong linear relationship with TiS_2_ concentration (Fig. S13). For pristine TiS_2_, the UV absorption peak intensity decreased rapidly within 2 h and finally disappeared in 24 h (left part of Fig. 3f), indicating a fast reaction of TiS_2_ nanosheets with water molecules, while the UV absorption peak intensity of DD-TiS_2_ nanosheets dispersion remained at 87% of its original intensity after standing for 24 h (right part of Fig. 3f), demonstrating that DDAB provides highly effective passivation, preventing oxidation and hydrolysis of TiS_2_ in aqueous environments. The enhanced antioxidant performances of these quaternary ammonium molecules on TiS_2_ membranes were also confirmed by atmospheric water vapor exposure experiment (Fig. 3g) and water immersion experiments (Fig. S14). The corresponding quantitative oxidation kinetics are summarized in Fig. S15, further confirming that water-induced hydrolysis is the dominant degradation pathway for pristine TiS_2_. The XPS results show that after a 24-h exposure to atmosphere, the TiO_2_ composition on DD-TiS_2_ membrane surface remained minimal, while the pristine TiS_2_ membrane surface was fully oxidized into TiO_2_. Zeta potential tests show that DD-TiS_2_ nanosheets exhibited the most positive charge (+ 64.4 mV, Fig. 3h), implying that DD-TiS_2_ nanosheets possess the strongest colloid stability contributing to the preservation of a well-defined layered structure during the RO tests. We note, however, that the zeta potential primarily reflects colloidal dispersion stability—governing how uniformly the nanosheets restack during membrane fabrication—rather than chemical durability under RO operating conditions. The long-term RO stability of the functionalized membranes instead originates from the densely packed amphiphilic alkyl layer that sterically shields the TiS_2_ surface from direct water contact, an interpretation that is corroborated by the energetic, structural, and mechanical evidence presented in the following sections. Moreover, concentration-dependent and pH-dependent profiles in Fig. S16 reveal that surfactant modification significantly elevated TiS_2_ surface potentials. While pristine TiS_2_ remained negative across all pH levels and DT-TiS_2_ reached an isoelectric point at pH 5.5, the DD- and TD-modified samples maintained strongly positive, stable charges from pH 3 to 10. This persistence confirms the robustness of the interfacial ammonium layers and the superior aqueous stability of the functionalized nanosheets. The continuous passivation of quaternary ammoniums enabled the TiS_2_ nanosheets to form an effective hydrophobic layer, thereby isolating TiS_2_ nanosheets from direct contact with water molecules, as also confirmed by the contact angle tests (Fig. 3i). Here, the contact angle for pristine TiS_2_ membrane was 65°, while DD-TiS_2_ membrane showed the highest contact angle of 103°. We further examined the dynamic wetting behavior of the membranes through time-dependent static contact angle measurements: Pristine TiS_2_ underwent rapid hydrophilization with complete droplet spreading within 1 min, indicating strong water affinity at the bare TiS_2_ surface, whereas all ammonium -modified membranes retained stable droplet shapes over 5 min (Fig. S17). The strong initial water uptake by pristine TiS_2_ provides a kinetically favorable pathway for the subsequent water-induced surface oxidation observed on longer timescales, while the persistent hydrophobicity of the modified membranes suppresses this initial water–surface contact step. The contact angle values can also be tuned by adjusting the quaternary ammonium salt concentration (Fig. S18). The calculated hysteresis (Δ*θ* = *θ*_adv_ − *θ*_rec_ ≈ 17°) indicated a homogeneous and low‐adhesion surface favorable for dynamic water repellence (Fig. S19). The functionalization degree per TiS_2_ for DTAB, DDAB, and TDAB reached ~ 19.5, ~ 41.7, and ~ 44.4 at%, respectively, as confirmed by thermogravimetric analysis, see Fig. S20.

### Atomic and Electronic Origins of Antioxidant and Hydrophobic Properties

**Fig. 4:**
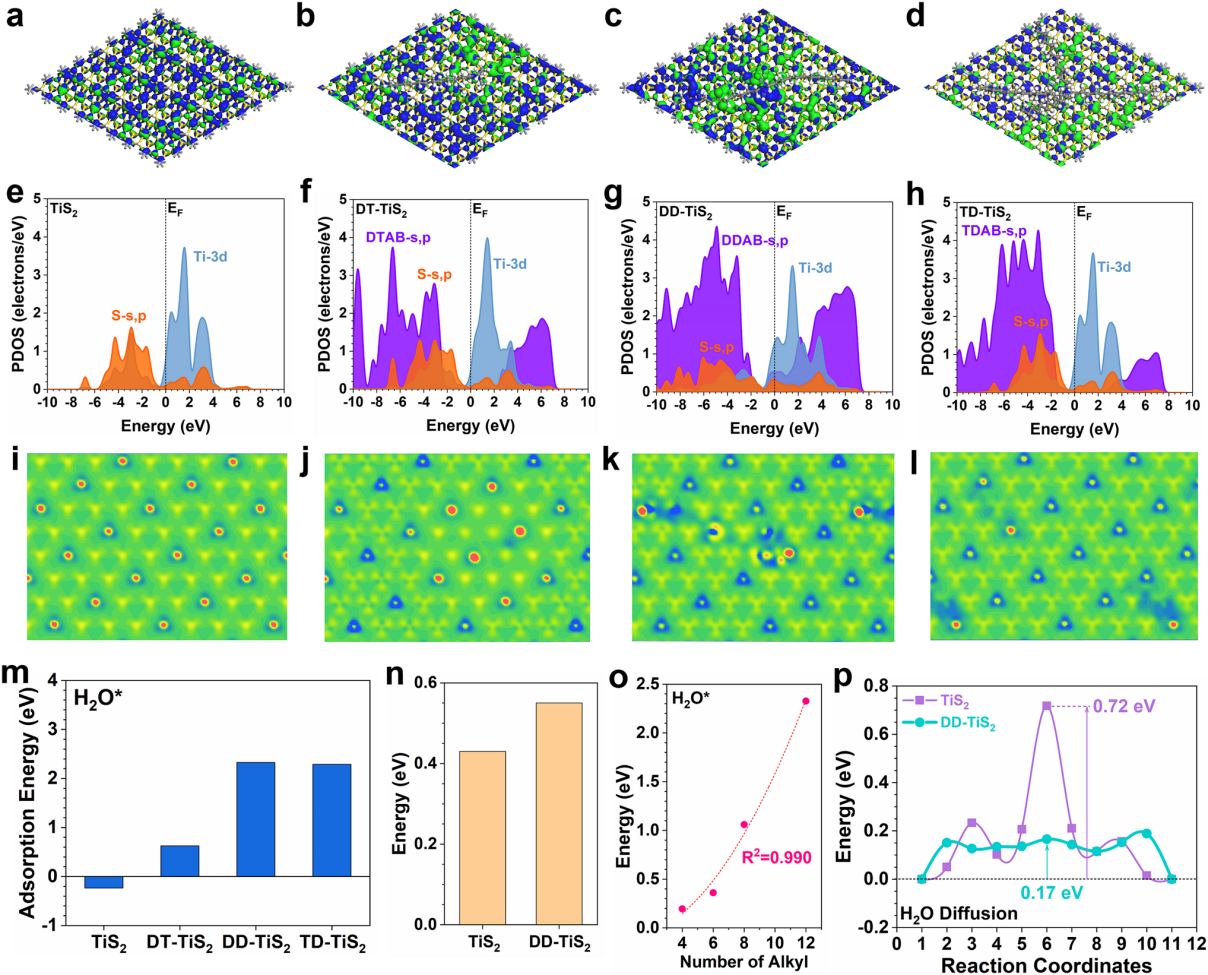
Electronic distributions near the Fermi level of **a** TiS_2_, **b** DT-TiS_2_, **c** DD-TiS_2_, and **d** TD-TiS_2_. Silver balls = Ti, yellow balls = S, dark gray balls = C, blue balls = N, and white balls = H. The blue isosurface = bonding orbitals, and the green isosurface = anti-bonding orbitals. The PDOS of **e** TiS_2_, **f** DT-TiS_2_, **g** DD-TiS_2_ and **h** TD-TiS_2_. In the electron density difference maps, red/yellow regions indicate electron accumulation, blue regions indicate electron depletion, and green regions represent relatively weak charge redistribution. The electron density difference of **i** TiS_2_, **j** DT-TiS_2_, **k** DD-TiS_2_ and **l** TD-TiS_2_. **m** Water adsorption energies on TiS_2_, DT-TiS_2_, DD-TiS_2_ and TD-TiS_2_. **n** Interaction energies with water for TiS_2_ and DD-TiS_2_. **o** Relationship between the adsorption energies of water and the number of alkyls. **p** Water diffusion barriers on TiS_2_ and DD-TiS_2_

To reveal the electronic modulations induced by long alkyl-chain surface modifications, we performed theoretical calculations. Surface electronic distributions near the Fermi level (E_F_) were first investigated in terms of pristine TiS_2_, DT-TiS_2_, DD-TiS_2_, and TD-TiS_2_ (Fig. 4a–d). Compared with pristine TiS_2_, the introduction of surface modifiers induces significant electronic redistribution at the interface. For pristine TiS_2_, continuous bonding orbitals are present near the E_F_, indicating higher electronic availability for interfacial charge transfer (Fig. 4a). After surface modification, the electronic states near E_F_ become more localized and partially redistributed due to interfacial interactions between the modifier molecules and surface S atoms. In contrast, the DT-TiS_2_, DD-TiS_2_, and TD-TiS_2_ show stronger distributions of the anti-bonding orbitals, which indicates the weakened surface capability of electron transfer and exchange (Fig. 4b–d). This suppresses the interactions with H_2_O for further oxidation. According to such electronic modulation, the intense order of DDAB on the surface of TiS_2_ will form a stable hydrophobic protection layer from both electronic structure and steric hindrance. To understand the surface electronic structures, we calculated the projected partial density of states (PDOS) (Fig. 4e–h). For pristine TiS_2_, the valence band maximum (VBM) is mainly dominated by hybridized Ti‑*3d* and S‑*3p* states, forming a delocalized electronic structure near the band edge (Fig. 4e). Such Ti‑derived contributions near VBM provide electronic states that can participate in interfacial redox reactions. Upon surface modification, the composition of the VBM progressively changes. For DT-TiS_2_, the *s,p* orbitals of the surface modifier are located at relatively deeper energy levels from E_F_ and are less directly involved in defining the VBM (Fig. 4f). For DD-TiS_2_ and TD-TiS_2_ systems, the molecular *s,p* states exhibit substantial overlap with Ti-*3d* and S-*3p* states near the VBM, indicating strong electronic hybridization at the interface (Fig. 4g, h). This compositional shift of the VBM from Ti/S-dominated states to partially molecule-influenced states reflects reduced Ti‑3*d* participation near the VBM, which is consistent with suppressed interfacial electron transfer tendency. To explore the interactions induced between DTAB/DDAB and TiS_2_, we demonstrated the electron density difference on the surface (Fig. 4i–l). Pristine TiS_2_ exhibits surface electron accumulation around S atoms, consistent with its negatively charged surface as reflected by the zeta potential measurements. After adsorption of DTAB, DDAB, and TDAB, the electron density near the surface S sites decreases due to electrostatic interaction with positively charged N^+^, leading to partial neutralization of surface charge. This trend agrees well with experimental zeta potential measurements, where DD‑TiS_2_ exhibits charge reversal and improved colloidal stability. These electronic structure analyses confirmed that surface modifications were able to significantly modulate the surface activity of the TiS_2_ nanosheets.

From the energetic perspective, we carried out detailed investigations to explore the interaction between modified TiS_2_ and water. For the adsorptions of water molecules, we noticed that only the pristine TiS_2_ surface shows negative adsorption energy, indicating thermodynamically favorable water adsorption (Fig. 4m). In contrast, DT‑TiS_2_, DD‑TiS_2_, and TD‑TiS_2_ exhibit positive adsorption energies, demonstrating energetically unfavorable water interaction with increasing hydrophobicity. This indicates that surface coverage and packing configuration collectively influence interfacial hydration behavior (Fig. 4n). To further simulate realistic aqueous environments, molecular dynamics (MD) simulations were performed by placing water droplets above pristine and modified TiS_2_ surfaces to evaluate interfacial structuring (Figs. S21 and S22). The density of water molecules in the near-surface region (~ 10 Å) is significantly reduced after surface modification when compared to TiS_2_, especially for DD‑TiS_2_, which is consistent with the contact angle results. In addition, we also investigated the interaction energies between pristine TiS_2_ and DD-TiS_2_ in water solution by MD simulations (Figs. 4k and S23a, b). Compared to pristine TiS_2_, the interaction with water requires higher energy costs for DD-TiS_2_, which further supports that the modified TiS_2_ is more hydrophobic with weaker interfacial affinity to water. Based on DD-TiS_2_, we systematically studied the number of alkyl groups to the hydrophobic property regarding the adsorption of water (Fig. 4o). Notably, as the alkyl chain becomes longer, the adsorption energy of water exhibits a good parabolic trend, where the correlation between alkyl number and adsorption energy reaches R^2^ = 0.990. In addition, to directly evaluate oxidation tendency, we calculated the adsorption energy of O_2_ on pristine and modified TiS_2_ (Fig. S24). Pristine TiS_2_ exhibits negative O_2_ adsorption energy, indicating a favorable interaction with oxygen molecules. In contrast, DT‑TiS_2_, DD‑TiS_2_, and TD‑TiS_2_ show positive adsorption energies with much reduced affinity. The simultaneously reduced affinity toward both H_2_O and O_2_ for modified TiS_2_ provides thermodynamic evidence that surface modification suppresses interfacial redox interactions, supporting the experimentally observed enhancement in oxidation resistance. Moreover, the water diffusion barriers were also determined on both TiS_2_ and DD-TiS_2_ (Fig. 4p). Water diffusion on DD-TiS_2_ is more facile, as indicated by the significantly reduced energy barrier of 0.17 eV. In contrast, the diffusion of water on pristine TiS_2_ is difficult with a high energy barrier of 0.72 eV, which is attributed to the strong adsorption strength of water on the surface. Thus, the DFT/MD results provide molecular-level evidence that alkyl-chain functionalization suppresses water–TiS_2_ interactions and stabilizes interlayer nanochannels, rather than directly predicting water permeance. It should be emphasized that the density-of-states and electron density difference analyses are used here to provide mechanistic insight into interfacial electronic tendencies, whereas the quantitative evidence for oxidation resistance is derived from the experimental XPS kinetics and time-resolved UV–Vis spectroscopy described above.

### Water Transport and Antibiotics Rejection

**Fig. 5:**
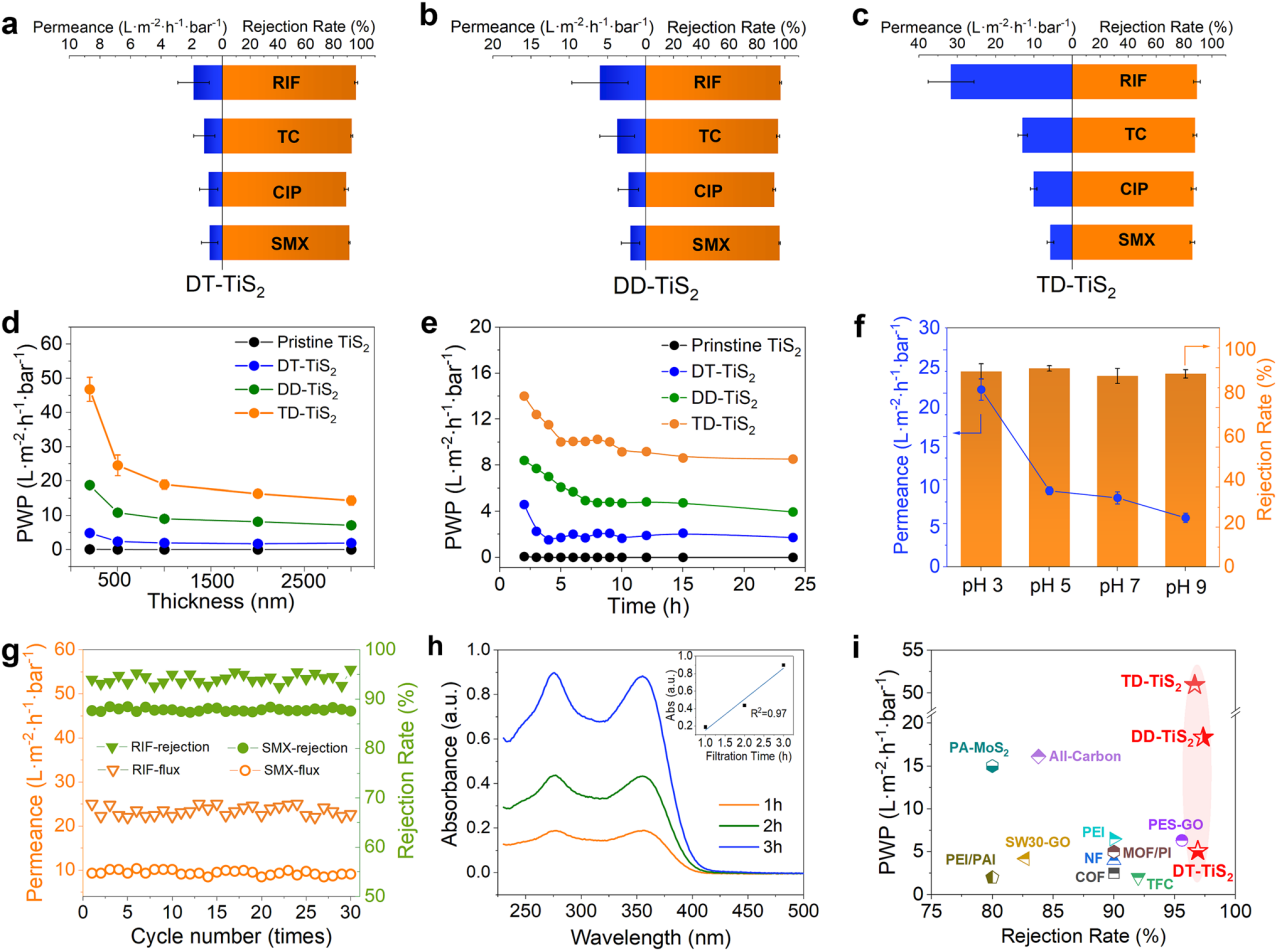
Separation performance and transport characteristics of functionalized TiS_2_ membranes. permeance and rejection of four representative antibiotics (RIF, TC, CIP, and SMX, 25 mg L^−1^, 25 °C) for **a** DT-TiS_2_, **b** DD-TiS_2_, and **c** TD-TiS_2_ membranes, respectively. A near-zero permeance for pristine TiS_2_ since pristine restacked TiS_2_ yields an effective capillary width of only ~ 0.02 nm, far smaller than the kinetic diameter of H_2_O (~ 0.27 nm), so no measurable transmembrane flux is obtainable under 6 bar. **d** Pure water permeance (PWP) of decorated membranes with thickness effect. All membranes were prepared under identical conditions: TiS_2_ nanosheet dispersions (0.5 mg mL^−1^ in DI water) were vacuum filtered onto PVDF substrates (220 nm pore size, 25 mm in diameter) at a constant vacuum of − 0.1 MPa and room temperature. Membrane thickness was tuned solely by adjusting the filtered volume (2–40 mL), yielding ~ 200 to 3000 nm thickness, respectively. For the functionalized membranes, 2 mL of 0.1 mM DTAB/DDAB/TDAB (ethanol/water = 1:1 v/v) was subsequently filtered through the freshly wet TiS_2_ layer under the same vacuum, followed by rinsing with 20 mL DI water. All samples were then vacuum-dried at 100 °C for 12 h. **e** Pure water permeability as a function of time for a membrane with a thickness of approximately 2 μm. **f** SMX permeance and rejection rate of DD-TiS_2_ in different pH feed solution. **g** Recycle properties of RIF and SMX using DD-TiS_2_ membrane. **h** UV spectra of TC solution (25 mg L^−1^, pH 6.5, 25 °C) on the retentate side with time. **i** Pure water permeance and rejection comparison of DT, DD, TD-TiS_2_ membranes and other reported membranes for antibiotics separation [47–56]. (Note that the pure water permeance (PWP) was measured using pure water, whereas the permeance values were obtained using antibiotic solutions.)

The antibiotics rejection performance of the functionalized TiS_2_ membranes was evaluated via a dead-end reverse osmosis system. The membrane diameter was 1 cm and the pressure at feed side was 6 bar (Fig. S25). A range of antibiotics (SMX, CIP, TC, and RIF) with varying molecular weights were tested (Fig. S26). The modified membranes exhibited simultaneously high water permeation rates and high rejection efficiencies toward all four antibiotics (Fig. 5a–c). The water permeation rate of above four antibiotics solution using DT-TiS_2_ membrane ranged from 0.8 to 1.8 L m^−2^ h^−1^ bar^−1^ (Fig. 5a); the results show a trend that antibiotics with larger molecular weights yielded higher permeation rates. This is because smaller-sized antibiotics were more effectively hindered by the sub-nanometer 2D capillaries. The permeation rates of DD-TiS_2_ membrane and TD-TiS_2_ membrane are approximately three times (2–6 L m^−2^ h^−1^ bar^−1^) and over ten times (5–31 L m^−2^ h^−1^ bar^−1^) higher than that of DT-TiS_2_ membrane, respectively (Fig. 5b, c), owing to their longer alkyl chains and larger interlayer spacing. Regarding rejection performance, DD-TiS_2_ membrane exhibited the highest rejection rates for the four sizes of antibiotic, with rejection rates of SMX, CIP, TC, and RIF being 96.3 ± 0.4%, 92.4 ± 1%, 95.1 ± 1%, and 96.9 ± 0.6%, respectively. For TD-TiS_2_ membrane, although permeation rates increased, the rejection rate of SMX decreased to 85.9%. This decrease is attributed to the fact that the capillary width of TD-TiS_2_ (~ 1.09 nm) significantly exceeds the molecular dimensions of SMX (MW = 253 g mol^−1^, the smallest antibiotic tested), rendering geometric confinement insufficient for complete exclusion. Consistent with the approximately cubic dependence of slit-channel flow on channel height, TD-TiS_2_ delivers the highest permeance (up to 46.8 L m^−2^ h^−1^ bar^−1^) among the three modified membranes, but the enlarged capillary also crosses the critical sieving threshold for SMX, leading to partial leakage. This exemplifies the classical permeance–selectivity trade-off for size sieving nanolaminate membranes: DD-TiS_2_ (capillary width  ~ 0.83 nm) represents the optimal balance between high flux and tight molecular confinement for all four antibiotics tested. Framing this as a quantitative structure–property relationship, the rejection of each antibiotic decreases when the capillary width δ exceeds the solute’s effective molecular dimension: For SMX (the smallest tested antibiotic molecule), DD-TiS_2_ (*δ* ~ 0.83 nm) just barely satisfies *δ* ≤ *d*_mol_ and retains 96.3% rejection, whereas TD-TiS_2_ (*δ* ~ 1.09 nm) crosses this cutoff and shows reduced rejection (85.9%). For the larger antibiotics (CIP, TC, RIF), the capillary width of TD-TiS_2_ remains below their molecular dimensions and rejection stays high. This establishes DD-TiS_2_ as the structure–property optimum among the three modifiers examined here. Figure 5d shows the thickness effect of the functionalized TiS_2_ membranes on pure water permeance (PWP); it can be observed that the water permeance decreased when increasing the membrane thickness from around 200 to 3000 nm (corresponding membranes’ thickness was determined by SEM images in Fig. S27), which is consistent with graphene oxide (GO) and other two-dimensional (2D)-based membranes. Note that the pure water permeance for DT-TiS_2_, DD-TiS_2_, and TD-TiS_2_ membranes was 4.3, 17.6, and 46.8 L m^−2^ h^−1^ bar^−1^, respectively, which were much higher than their unmodified counterpart, and TD-TiS_2_ membrane showed the largest variations on changing the membrane thickness. Figure 5e illustrates the changes in PWP of the functionalized TiS_2_ membranes during the initial 24 h of RO testing. The initial decline in flux was a typical start-up effect of RO membranes and did not contradict the lower diffusion barrier. It mainly arises from rapid compaction and progressive wetting of the membrane, which transiently increase hydraulic resistance. After reaching steady state, the DD-TiS_2_ membrane exhibited a consistently higher flux, aligning with the reduced energy barrier predicted by DFT. Although increased surface hydrophobicity might intuitively be expected to impede water transport, the net permeation behavior is dominated by the dramatic interlayer expansion: According to the Hagen–Poiseuille framework adapted for slit nanochannels, water flux scales approximately with the cube of channel height, so the order-of-magnitude increase in capillary width (0.02 → 0.37 → 0.83 → 1.09 nm) overwhelms any resistance that might arise from hydrophobic wetting. Moreover, the hydrophobic surface may reduce water–wall friction and facilitate a slip-flow contribution, which could further support the enhanced permeance, consistent with the markedly reduced water diffusion barrier (from 0.72 to 0.17 eV) established by our DFT calculations above. The wettability and channel geometry effects therefore act synergistically, rather than antagonistically, to enhance water permeance. Among, TD-TiS_2_ membrane maintained the highest water flux, due to the largest functionalization molecules TDAB. Furthermore, we investigated the pH effect to rejection rate and water flux for DD-TiS_2_ membrane. The permeance (Fig. 5f) shows sensitivity to pH—likely due to slight changes in membrane surface hydrophilicity or protonation state—the rejection performance remains stable. The quaternary ammonium headgroups (R_4_N^+^) on the DD-TiS_2_ surface carry a permanent, pH-independent positive charge, whereas sulfamethoxazole (SMX) exhibits two acid dissociation constants, with pKa_1_ ≈ 1.7 for the aniline amino group and pKa_2_ ≈ 5.7 for the sulfonamide N–H group. Accordingly, SMX is mainly neutral at mildly acidic to near-neutral pH, with limited cationic character only under strongly acidic conditions, and becomes predominantly anionic above pH 5.7. Electrostatic repulsion may therefore assist SMX rejection under acidic conditions, whereas electrostatic attraction could occur under alkaline conditions. The stable rejection (> 95%) across pH 3–10 confirms that geometric size sieving through the sub-nanometer nanochannels is the dominant separation mechanism, while electrostatic interactions serve only as a secondary, pH-dependent modulation.

### Structural Stability and Long-Term Operational Durability

For the long-term recyclability and stability evaluation of the membrane materials, the mechanical properties were tested first. The flexibility and structural integrity of the TiS_2_-based membranes under repeated bending tests were first examined. As shown in Fig. S28a, the membranes were pre-folded several times to simulate the mechanical deformation experienced during cyclic reuse and under RO operating pressure. Corresponding SEM observations after repeated folding (Fig. S28b-d) further confirmed that the modified membranes preserved a continuous and compact lamellar morphology, with no observable fracture planes or peeling of nanosheets. In addition, cross-sectional SEM images after repeated folding are added as Fig. S28e-j (analogous to the cross-sectional views of fresh membranes shown earlier) to directly visualize the preserved lamellar architecture after mechanical deformation. Mechanical tensile tests (Fig. S29) revealed that quaternary ammonium-functionalized membranes exhibited significantly enhanced mechanical strength compared with pristine TiS_2_. Both DT-TiS_2_ and DD-TiS_2_ showed higher tensile stress and improved elongation at break, which we attribute to stronger interfacial interactions between TiS_2_ nanosheets and the polymer substrate, as well as increased interlayer friction introduced by the amphiphilic modifiers. The mechanical enhancements corroborated that surface functionalization not only stabilized TiS_2_ chemically but also reinforced the structural integrity of the laminated architecture, ensuring reliable performance during repeated handling and long-term membrane operation. Specifically, these “interfacial interactions” include the electrostatic attraction between the cationic ammonium headgroups and the anionic TiS_2_ surface, the hydrophobic–hydrophobic adhesion between the amphiphilic modifier tails and the underlying PVDF substrate, and the increased interlayer friction introduced by the interdigitated alkyl chains, which collectively enhance both tensile strength and elongation at break.

To evaluate the structural stability of the membranes, we analyzed the elemental composition before and after RO operation. There was no detectable shift in the N 1*s* peak position or shape before and after RO operation, with XPS semi-quantitative analysis showing < 1% change in N atomic content, indicating no obvious degradation or loss of the quaternary ammonium functional layer during long-term filtration (Fig. S30). We quantitatively analyzed the Ti 2*p* spectra to determine TiS_2_/TiO_2_ ratios before and after RO (Fig. S31). The pristine TiS_2_ membrane underwent complete oxidation (65% TiS_2_ → 100% TiO_2_), whereas the functionalized membranes retained substantially higher TiS_2_ contents (DT-TiS_2_: 60% → 54%, DD-TiS_2_: 63% → 61%, TD-TiS_2_: 73% → 32%). These results clearly indicated that DD-TiS_2_ provided the most effective oxidation protection, while the reduced stability of TD-TiS_2_ likely arises from steric hindrance associated with its longer alkyl chains, which weakens organic–inorganic interfacial bonding and allows partial oxidation during operation. The XRD patterns of DT-TiS_2_ and DD-TiS_2_ membranes remained essentially unchanged after RO, whereas TD-TiS_2_ exhibited a noticeable right shift of the (001) TiS_2_ reflection—indicative of reduced interlayer spacing and partial structural collapse. This degradation was consistent with the weaker bonding between the long-chain modifier and TiS_2_, as well as the partial oxidation revealed by XPS (Fig. S32). The total organic carbon (TOC) levels of all permeates were close to that of DI water, confirming that no measurable leaching of quaternary ammonium modifiers occurred (Fig. S33).

For the cyclic stability evaluation, 30 filtration cycles and a 15 days continuous operation test were conducted for these four kinds of antibiotics. The optimized DD-TiS_2_ membrane retained stable rejection (RIF, TC, and SMX above 91%; CIP above 89%) across 30 filtration cycles and 15 days of continuous operation (each cycle included the process of pressure release and membrane recovery with DI water), implying good stability of the prepared membrane, as shown in Figs. 5g and S34-S35. Note that pristine TiS_2_ membranes were not included as a cycling control because the interlayer spacing of restacked pristine TiS_2_ (0.57 nm) yields an effective capillary width of merely ~ 0.02 nm—far smaller than the kinetic diameter of a water molecule (~ 0.27 nm)—resulting in essentially no measurable transmembrane water flux under the 6 bar operating pressure, which precludes reliable permeance or rejection measurements over repeated filtration cycles. Figure 5h shows the TC concentration on the retentate side of the DD-TiS_2_ membrane increasing linearly over time (via UV–Vis), confirming that size sieving is the predominant mechanism rather than membrane adsorption. Furthermore, the adsorption of all four antibiotics (SMX, CIP, TC, and RIF) onto TiS_2_ nanosheets was confirmed to be negligible (Fig. S36). This stability across conditions underscores that physical confinement, not surface charge or adsorption, dictates the superior separation performance. The theoretical calculations presented above reveal the electronic origins of the enhanced oxidation resistance of the functionalized TiS_2_ surface, whereas the antibiotic separation itself is governed by geometric confinement within the sub-nanometer capillaries; these two findings are therefore complementary rather than contradictory. For DD-TiS_2_ membrane, SEM images showed the accumulation of four antibiotics on membrane surface after RO testing (Fig. S37); EDS mapping results indicated a significant increase in carbon content after RO test, which was attributed to the rejected antibiotics that remaining on the feed side (Fig. S38). AFM image analysis revealed similar surface roughness, demonstrating the integrity of the membrane without cracks (Fig. S39). Compared with the existing literature, our quaternary ammonium molecules modified TiS_2_ membranes (especially DD-TiS_2_ membrane) exhibited best rejection rate (96.9%) and permeance (46.8 L m^−2^ h^−1^ bar^−1^) for potential practical application in medical wastewater treatment (Fig. 5i, Table S1).

The economic assessment, based on a pouch cell-style scalable production model (Fig. S40), shows that the dominant cost contribution (> 75%) arises from the TiS_2_ raw material itself, whereas the quaternary ammonium modifiers, PVDF substrate, and processing steps account for only a minor portion of the overall production cost. The Sankey diagram further confirmed that operational cost overwhelmingly outweighs fixed and capital costs, indicating that the fabrication route—electrochemical exfoliation followed by vacuum filtration—was not a cost-limiting step. Because all reagents involved are industrially available and no high-temperature or high-pressure conditions were required, the overall process was compatible with scale-up. As shown in Fig. S41, conventional liquid-phase exfoliation of graphene typically requires ~ 20–30 kWh kg^−1^, while chemical exfoliation of layered MoS_2_ can exceed ~ 30 kWh kg^−1^. Even advanced low-energy processes such as mechanical shear exfoliation or rapid Joule heating (RJH) graphene still consume ~ 4.8 kWh kg^−1^. In contrast, the pouch cell-assisted exfoliation route presented in this work requires only ~ 0.72 kWh kg^−1^ (based on Tables S2 and S3), owing to the intrinsically mild processing conditions and the absence of high-temperature or high-shear operations. These results demonstrated that the proposed membrane preparation method had a clear economic feasibility and aligns with the cost structure reported for large-scale soft-pack battery manufacturing. The additional cross-flow filtration tests (Fig. S42) yielded separation results comparable to those of the dead-end mode, further validating the membrane's robust stability and promising potential for practical, large-scale applications. To assess membrane performance under model feed conditions that partially approximate practical wastewater matrices, we further examined the rejection of all four antibiotics (RIF, TC, CIP, SMX) in mixed-solute feeds (each at 25 mg L^−1^) under varying ionic strength backgrounds to represent ionic strength effects in practical wastewater, using both monovalent (NaCl, 1–10 mM) and divalent (CaCl_2_, 1–10 mM) electrolytes. As shown in Fig. S43, the DD-TiS_2_ membrane retains rejection above 90% for all four antibiotics across the entire tested range, with only minimal sensitivity to ionic strength and a slightly larger perturbation under CaCl_2_ than under NaCl for the smaller antibiotics—consistent with divalent cation screening of the cationic surface charge. The relative ranking of the three membranes (DD-TiS_2_ > DT-TiS_2_ > TD-TiS_2_ for the smaller antibiotics) is preserved across all conditions, further confirming that geometric size sieving remains the dominant separation mechanism. This study mainly focuses on the effect of membrane ion channels on solute rejection under single-variable conditions in a pure water system; radical-mediated pathways caused by dissolved oxygen, residual chlorine/ozone from pretreatment, or trace metal ions under actual RO influent conditions may introduce additional degradation mechanisms and deserve further study.

## Conclusion

In conclusion, we developed an emerging titanium disulfide (TiS_2_)-based nanosheet with a stable 1T phase for fabricating high-performance nanolaminate membranes. These membranes featured precisely tunable sub-nanometer capillary widths, enabled by a rapid and universal electrostatic functionalization strategy using a group of quaternary ammonium molecules (DTAB, DDAB, TDAB). The functionalized TiS_2_ membranes exhibited significantly enhanced surface hydrophobicity and markedly improved resistance to water-induced oxidation, leading to exceptional structural robustness and sustained operational stability over the tested duration. Notably, the membranes demonstrated remarkable rejection rates up to 96.9% for various antibiotics, with a permeance up to 46.8 L m^−2^ h^−1^ bar^−1^. These results contribute significantly toward the efficient and reliable removal of emerging small-molecule pollutants from water bodies.

## Supplementary Information

Below is the link to the electronic supplementary material.

Supplementary file1 (DOCX 26102 kb)
