# Supplementary material for: Enhanced Antibiotics Sieving by Exfoliated TiS2 Membranes via Surface Functionalization and Passivation
Source: Nanomicro Lett. 2026 Aug 14;19:34. doi: 10.1007/s40820-026-02315-4 (PMC13473043; doi:10.1007/s40820-026-02315-4)
Supplement: Supplementary file 1 [file 40820_2026_2315_MOESM1_ESM.docx]

Supporing Information for

**Enhanced Antibiotics Sieving by Exfoliated TiS_2_ Membranes via Surface Functionalization and Passivation**

Ruixin Yan^1^†, Mingzi Sun^2^†, Honglu Hu^1^, Ruijie Yang^1^, Zhen Zhang^1^, Weikang Zheng^1^, Liang Mei^1^, Ting Ying^1^, Yue Zhang^1^, Alicia Kyoungjin An^3^, Chuyang Y. Tang^4^, Jingyun Fang^5^*, Bolong Huang^2^*, Zhiyuan Zeng^1,6^*

^1^Department of Materials Science and Engineering, and State Key Laboratory of Marine Environmental Health, City University of Hong Kong, 83 Tat Chee Avenue, Kowloon, Hong Kong S.A.R. 999077, P. R. China

^2^Department of Chemistry, City University of Hong Kong, Hong Kong S.A.R. 999077, P. R. China.

^3^Department of Chemical and Biological Engineering, The Hong Kong University of Science and Technology, Clear Water Bay, Kowloon, Hong Kong S.A.R. 999077, P. R. China

^4^Department of Civil Engineering, The University of Hong Kong, Hong Kong S.A.R. 999077, P. R. China

^5^Guangdong Provincial Key Laboratory of Environmental Pollution Control and Remediation Technology, School of Environmental Science and Engineering, Sun Yat-Sen University, Guangzhou 510275, P. R. China

^6^Shenzhen Research Institute, City University of Hong Kong, Shenzhen 518057, P. R. China

†Ruixin Yan and Mingzi Sun contributed equally to this work.

*Corresponding authors. E-mail: zhiyzeng@cityu.edu.hk (Zhiyuan Zeng); b.h@cityu.edu.hk (Bolong Huang); fangjy3@mail.sysu.edu.cn (Jingyun Fang)

**Supplementary Figures**

**Fig. S1** SEM images of commercial TiS_2_ powder and their elemental mapping distribution.

**Fig. S2** Chemical structure of three quaternary ammonium molecules: DTAB, DDAB, TDAB.

**Fig. S3** a) TEM images and elemental mapping of pristine TiS_2_ nanosheets. b) Raman spectra of bulk and exfoliated TiS_2_ nanosheets.

**Fig. S4** XPS image of bulk TiS_2_ powders and exfoliated TiS_2_ nanosheets.

**Fig. S5** SEM EDS mapping images of pristine TiS_2_ membrane. (The porous region corresponds to the PVDF substrate, while the tightly packed layered structure directly above it represents the restacked film.)

**Fig. S6** XPS spectra of a) N1s of membranes of TiS_2_/DT-TiS_2_/DD-TiS_2_/TD-TiS_2_ and DTAB/DDAB/TDAB powders. b, c) Ti2p and S2p of membranes of TiS_2_/DT-TiS_2_/DD-TiS_2_/TD-TiS_2_. From the XPS spectra before and after the reaction, changes in the binding energies of surface elements can be observed. The N 1s peaks show the presence of ammonium peaks on the surface of TiS_2_ modified with quaternary ammonium salts, corresponding to the N positions in the three modifiers' powders. This indicates that the positively charged ammonium ends have successfully attached to the TiS_2_ surface [S1, S2]. The N peak on the unmodified TiS_2_ surface may originate from nitrogen in the air, with adsorption sites competing with quaternary ammonium ions. After modification, its relative content decreases. From the Ti 2p spectrum, the peaks for TiS_2_ (456 eV) and TiO_2_ (459 eV) can be directly identified [S3, S4]. It can be observed that pure TiS_2_ shows a significant number of oxides during the exfoliation and drying process. In contrast, the samples protected by a quaternary ammonium salt hydrophobic layer maintain a TiS_2_ content similar to the original powder. Correspondingly, the S 2p spectrum shows only one pair of peaks, indicating that no phase transformation occurred during the exfoliation and modification process. d) EDS mapping of DD-TiS_2_ nanosheets.

**Fig. S7** Optical images of four kinds of modified membranes.

**Fig. S8** Raman shift of DTAB/DDAB/TDAB modified TiS_2_ membrane. The Raman shift of bulk TiS_2_, identified as the 1T polytype, exhibits two distinct active modes: A_1g_ and E_g_, positioned at approximately 332 cm^-1^ and 230 cm^-1^, respectively [S5]. The A_1g_ mode is characterized by the out-of-plane vibrations of sulfur atoms, while the E_g_ mode encompasses both in-plane and out-of-plane vibrations of sulfur atoms. The A_1g_ mode remains largely unchanged, indicating that the modified TiS_2_ membrane has retained its 1T phase without significant phase transformation. The E_g_ mode in the modified membrane exhibits a degree of broadening, which could likely be attributed to the surface modification by quaternary ammonium molecules.

**Fig. S9** FTIR images of a) commercial DTAB/DDAB/TDAB/bulk TiS_2_ powders. b) pristine and modified TiS_2_ membranes. A strong absorption of the -CH_2_- peak was found in the 2800-3000 cm^-1^ range and 720 cm^-1^ for all three modifiers [S6]. After reaction with TiS_2_ nanosheets, excepts the pristine TiS_2_, all TiS_2_ membranes modified with the three quaternary ammonium salts show enhanced absorption of methylene groups, this indicates the successful grafting of alkyl chains on the surface of the membranes.

**Fig. S10** SEM images of membrane thickness changes before and after DDAB modification. (The porous region corresponds to the PVDF substrate, while the tightly packed layered structure directly above it represents the restacked film.)

**Fig. S11** Structural and morphological comparison between pristine and oxidized TiS_2_. a) AFM image of a pristine TiS_2_ nanosheet, showing a smooth and intact surface morphology. b) TEM image of pristine TiS_2_, revealing a continuous lattice without observable oxidation-induced defects. c) Cross-sectional SEM image of a pristine TiS_2_ membrane, displaying well-defined and orderly lamellar stacking. d) XRD pattern of the pristine TiS_2_ membrane, characterized by a sharp (001) reflection, indicative of preserved layered crystallinity. e) AFM image of an oxidized TiS_2_ nanosheet, where multiple oxidation-induced voids are observed (highlighted by white dashed circles). f) TEM image of oxidized TiS_2_, showing pronounced voids and contrast variations associated with oxidation (marked by red dashed circles). g) Cross-sectional SEM image of the oxidized TiS_2_ membrane, exhibiting disrupted lamellar stacking and partial structural collapse after oxidation. h) XRD patterns of TiS_2_ membranes after 12 h and 24 h RO operation, showing weakened and broadened diffraction features, suggesting a gradual loss of layered order upon oxidation.

**Fig. S12** Morphology and thickness characterization of functionalized TiS_2_ nanosheets. a–c) TEM images of DT-TiS_2_ (a), DD-TiS_2_ (b), and TD-TiS_2_ (c) nanosheets. d–f) AFM topographic images of DT-TiS_2_ (d), DD-TiS_2_ (e), and TD-TiS_2_ (f). g–i) Corresponding AFM height profiles extracted along the indicated lines, showing average thicknesses of ~3 nm for DT-TiS_2_ (g), ~4 nm for DD-TiS_2_ (h), and ~6 nm for TD-TiS_2_ (i).

**Fig. S13** a) UV-vis spectra absorbance of TiS_2_ ink solution. b) linear relationship between TiS_2_ concentration and absorbance.

**Fig. S14** This presented a significant color change from the metallic luster of TiS_2_ before reverse osmosis, directly illustrating the water sensitivity of the TiS_2_ membrane. Notably, the surfaces of membranes modified with three types of DTA ions maintained their original metallic luster even after the reverse osmosis test, directly confirming the significant anti-oxidative effect of DTA ions on TiS_2_ films. The immersion experiments were also conducted in water sink that further validate the protective effect of DTA ions on TiS_2_. As shown, the unmodified TiS_2_ membrane exhibits significant cracking and discoloration after 48 hours of immersion, and these damaged areas pose a potential risk of secondary contamination during practical applications. In contrast, the modified TiS_2_ membrane, which remains stable after modification, not only demonstrates excellent antioxidation properties but also exhibits some degree of swelling inhibition. This remarkable effect is largely due to the rapid electrostatic forces at play during the post-treatment process, which enable the DTA ions to swiftly self-assemble. This results in the N^-^terminal end aligning closely with the TiS_2_ side, while the hydrophobic long chains orient towards the water molecules.

**Fig. S15** Quantitative oxidation kinetics of pristine TiS_2_ under three representative conditions: water immersion (blue, derived from time-resolved UV-vis absorbance decay), ambient air drying at 25 °C (green, derived from time-resolved Ti 2p XPS deconvolution), and forced air drying at 60 °C (orange, derived from time-resolved Ti 2p XPS deconvolution). The comparison demonstrates that hydrolysis by H_2_O, rather than oxidation by molecular O_2_, is the dominant degradation pathway of TiS_2_.

**Fig. S16** Zeta potential evolution of pristine and ammonium-modified TiS_2_ nanosheets. a–c) Zeta potential distributions of TiS_2_ modified with increasing amounts of DTAB (a), DDAB (b), and TDAB (c). Pristine TiS_2_ exhibits a strongly negative surface potential (≈ –50 to –60 mV). Progressive addition of the quaternary ammonium salts leads to a systematic shift toward positive values, indicating successful adsorption of the cationic headgroups and formation of a surface-stabilizing layer. Complete charge reversal to +70~100 mV is observed at sufficiently high modifier concentrations. d) pH-dependent zeta potentials of pristine TiS_2_ and the three modified TiS_2_ samples. While pristine TiS_2_ becomes unstable and approaches neutral/negative charge across the pH range, all modified samples maintain strongly positive and relatively stable surface potentials from pH 3 to 10, demonstrating the robustness of the interfacial ammonium layers and improved aqueous stability.

**Fig. S17** Time‐dependent static contact angles of pristine TiS_2_, DT‐TiS_2_, DD‐TiS_2_, and TD‐TiS_2_ membranes, showing distinct wetting evolution from 0 ~ 5 min. Pristine TiS_2_ rapidly loses hydrophobicity, whereas modified membranes retain high contact angles (>85°) over time.

**Fig. S18** Contact angle of membranes decorated by different concentrations (40 μL, 80 μL, 160 μL) of DTAB/DDAB/TDAB.

**Fig. S19** Advancing and receding contact angle measurements on a DT‐TiS_2_ membrane under tilting angles of 10° and 50°. The calculated hysteresis (Δθ = θ_adv_ − θ_rec_ = 17°) indicates a homogeneous and low‐adhesion surface favorable for dynamic water repellence.

**Fig. S20** TGA of bulk TiS_2_ powders and DT/DD/TD-TiS_2_.

**Fig. S21** MD simulations of water droplet before and after interactions with pristine and modified TiS_2_ surfaces. (a, b) TiS_2_, (c, d) DT-TiS_2_, (e, f) DD-TiS_2_, and (g, h) TD-TiS_2_. Color bars: Silver balls = Ti, Yellow balls = S, Red balls = O, Grey balls = C, white balls = H. The top blue balls are inert atoms as the block for water molecules during the MD simulations^1-5^.

**Fig. S22** Ratio of water molecules from the droplets located near the surface of pristine and modified TiS_2_ surfaces.

**Fig. S23** The MD simulation models of (a) DD-TiS_2_ and (b) TiS_2_ interact with water molecules in the solutions at different angles.

**Fig. S24** Adsorption energies of O_2_ on TiS_2_, DT-TiS_2_, DD-TiS_2_, and TD-TiS_2_.

**Fig. S25** The image of RO test system. A homemade dead-end filtration cell was used to evaluate membrane permeance under nitrogen pressure. The feed solution was loaded into the stainless-steel filtration cell and pressurized by nitrogen gas applied from the top. The membrane was placed at the bottom of the cell, and the permeate solution was collected through the outlet tubing into a graduated cylinder for volume measurement. The entire setup was placed on a magnetic stirring plate to minimize concentration polarization during filtration.

**Fig. S26** Chemical structure of four kinds of antibiotics and their molecular weight.

Fig. S27 Cross-sectional SEM images of pristine and functionalized TiS_2_ membranes with tunable thicknesses. All membranes were prepared under identical conditions: TiS_2_ nanosheet dispersions (0.5 mg mL^−1^ in DI water) were vacuum-filtered onto PVDF substrates (220 nm pore size, 25 mm in diameter) at a constant vacuum of −0.1 MPa and room temperature. Membrane thickness was tuned solely by adjusting the filtered volume (2, 5, 10, 20, or 40 mL), yielding ~200, ~600, ~1000, ~2000, and ~3000 nm stacks, respectively. For the functionalized membranes, 2 mL of 0.1 mM DTAB/DDAB/TDAB (ethanol/water = 1:1 v/v) was subsequently filtered through the freshly wet TiS_2_ layer under the same vacuum, followed by rinsing with 20 mL DI water. All samples were then vacuum-dried at 100 °C for 12 h. (scale bar: 2 μm)

**Fig. S28** Mechanical flexibility and tensile performance of TiS_2_-based membranes. a) Optical photograph showing the free-standing DT-TiS_2_ membrane bent into a semicircular shape without visible cracks or delamination, demonstrating excellent flexibility. b–d) SEM images of the membrane surface after bending, confirming continuous and compact morphology for DT-TiS_2_, DD-TiS_2_, and TD-TiS_2_ samples without structural failure. e-j) Cross-sectional SEM images of e) DT-TiS_2_, f) DD-TiS_2_, and j) TD-TiS_2_ membranes before (top row) and after (bottom row) bending. The tightly packed, layered internal structures remain fully intact, further verifying their excellent structural integrity under mechanical deformation.

**Fig. S29** Stress–strain curves of PVDF, pristine TiS_2_, and modified TiS_2_ membranes (DT-TiS_2_, DD-TiS_2_, TD-TiS_2_), showing improved tensile strength (2.8–3.1 MPa) and good elongation (30–45%) for all modified membranes.

**Fig. S30** XPS N 1s spectra of functionalized TiS_2_ membranes before and after RO tests. a) Fresh DT-TiS_2_ membrane. b) DT-TiS_2_ membrane after RO test. c) Fresh DD-TiS_2_ membrane. d) DD-TiS_2_ membrane after RO test. e) Fresh TD-TiS_2_ membrane. f) TD-TiS_2_ membrane after RO test.

**Fig. S31** Ti 2p XPS spectra of pristine and modified TiS_2_ membranes before and after RO operation. a–d) High-resolution Ti 2p spectra of fresh TiS_2_, DT-TiS_2_, DD-TiS_2_, and TD-TiS_2_ membranes. e–h) Corresponding Ti 2p spectra after RO tests. The blue peaks correspond to Ti^4^⁺ (TiS_2_) and the yellow peaks to Ti^4^⁺ (TiO_2_) species. The integrated areas of these deconvoluted peaks were used to evaluate the TiS_2_/TiO_2_ ratio, reflecting the degree of surface oxidation before and after RO operation.

**Fig. S32** XRD patterns of a) DT-TiS_2_, b) DD-TiS_2_, and c) TD-TiS_2_ membranes before and after RO operation.

**Fig. S33** Total organic carbon (TOC) concentrations of permeate water after filtration through different membranes.

**Fig. S34** Cyclic stability of DD-TiS_2_ membranes in antibiotic rejection tests. Permeance (orange, left axis) and rejection rate (green, right axis) of DD-TiS_2_ membranes tested over 30 consecutive filtration cycles for a) CIP, b) TC (25 mg/L, 25°C).

**Fig. S35** Long-term operation stability of DD-TiS_2_ membranes. (RIF, TC, CIP, and SMX, 25 mg/L, 25 °C)

**Fig. S36** UV-vis absorbance before and after adsorption of four kinds of antibiotics.

**Fig. S37** SEM images of RO-tested DD-TiS_2_ membrane.

**Fig. S38** SEM EDS mapping of a) fresh DD-TiS_2_ membrane and b) RO-tested DD-TiS_2_ membrane.

**Fig. S39** AFM images of DD-TiS_2_ membranes after RO test of SMX/CIP/TC/RIF.

**Fig. S40** Techno-economic assessment of TiS_2_ nanosheet production via the soft-pack battery exfoliation route. a) Cost distribution of producing 1 kg TiS_2_ nanosheets, showing total cost ≈ $700 kg⁻^1^, dominated by TiS_2_ and LiFePO4 raw materials. b) Sankey diagram detailing the cost flow from raw materials, capital, and utilities to individual components. Operational and capital expenses contribute < 0.05%, confirming that the exfoliation and filtration processes are economically scalable. Abbreviations: FCI, fixed capital investment; FCOP, fixed costs of production; UTS, total utilities; CONS, total consumables. c) optical image of pouch-cell using TiS_2_ as electrode for scalable production.

**Fig. S41** Comparison of fabrication energy consumption for different membrane and 2D-material production routes. Energy consumption (kWh·kg⁻1 of material) for several representative fabrication processes, including liquid-phase exfoliation of graphene, chemical exfoliation of MoS_2_, mechanical shear exfoliation of graphene, rapid Joule-heating (RJH) graphene, graphene obtained from waste precursors, and polymeric blend RO membrane fabrication (literature values).

**Fig. S42** Cross-flow filtration performance of the DD-TiS_2_ membrane. a) Photograph of the laboratory-scale cross-flow filtration setup used in this study. b) Optical images of the DD-TiS_2_ membranes (effective area ≈ 2 cm^2^) installed in the stainless-steel cross-flow module. c) Permeance and rejection of four representative antibiotics (SMX, CIP, TC, and RIF at a concentration of 25 ppm, 25°C) under cross-flow operation.

**Fig. S43** Antibiotic rejection performance of the three functionalized TiS_2_ membranes in mixed-antibiotic feeds (RIF + TC + CIP + SMX, each at 25 mg/L) under varying ionic-strength backgrounds. a–c) Rejection rates in NaCl backgrounds of 1, 2, 5, and 10 mM for a) DT-TiS_2_, b) DD-TiS_2_, and c) TD-TiS_2_ membranes. d–f) Corresponding rejection rates under CaCl_2_ backgrounds of 1, 2, 5, and 10 mM for d) DT-TiS_2_, e) DD-TiS_2_, and f) TD-TiS_2_ membranes. All measurements were performed under dead-end RO operation at 6 bar transmembrane pressure with a 1 cm effective membrane diameter. DD-TiS_2_ retains the highest rejection (>88% for all four antibiotics) across the entire ionic-strength range in both monovalent and divalent backgrounds, demonstrating robust performance in mixed-solute feeds.

**Table S1** Summary of the membrane materials, filtration modes, and operating conditions of the reference works compared in **Fig. 5i**

| **Membrane** | **Filtration Mode** | **Operating Pressure** | **Target Solute & Concentration** | **Ref.** |
| --- | --- | --- | --- | --- |
| PES-GO | Dead-end | 3 bar | Reactive Red 195, 100 mg/L; BSA, 250 mg/L | [S7] |
| NF (PEI/PI) | Dead-end | 13.8 bar | Sulfamerazine, 1 mg/L | [S8] |
| PA-MoS_2_ | Cross-flow | 8 bar | Four parabens (methyl-, ethyl-, propyl-, benzyl-paraben), 200 μg/L each | [S9] |
| MOF/PI | Cross-flow | 5–11 bar | 21 PhACs, 20 μg/L each; NaCl and MgSO_4_, 2000 mg/L each | [S10] |
| SW30-GO | Cross-flow | 400 psi | NDMA, 890 μg/L | [S11] |
| PEI | Dead-end | 13.8 bar | Cefadroxil, 1 mg/L; Enrofloxacin, 1 mg/L | [S12] |
| COF | Cross-flow | 5 bar | Five dyes (chrome black T, methyl blue, Congo red, acid fuchsin, rose bengal), 100 mg/L | [S13] |
| TFC | Dead-end | 5 bar | MR, MnB, RosB, BB, 10 mg/L each | [S14] |
| PEI/PAI | Cross-flow | 10 bar | Ciprofloxacin, 1 ppm | [S15] |
| All-carbon | Vacuum filtration | 0.9 bar | Tetracycline hydrochloride (TCH), 500 μM, 20 mL | [S16] |

**Table S2** Energy flow data

| Parameter | Value | Unit | Ref |
| --- | --- | --- | --- |
| Battery | -2800 | kW | = – (industrial-scale output / laboratory-scale output) × (battery power × discharge time) |
| Ultrasonic bath | 418 | kW | = Ultrasonic bath scale* unit volumetric energy consumption of the ultrasonic bath |
| Centrifuge | 256 | kW | a |
| Mixer | 47 | kW | =15*Mixer scale^1[S17] |

a：The power consumption of the centrifuge is calculated according to Ref [S18]. As:

$$Centrifuge Power \left( W \right)= \omega^{2}（m_{s}r_{DS}^{2}+m_{L}r_{DL}^{2}）$$

r_DS_=0.21 m，r_DL_=0.285 m

m_S_(solid feed rate) =0.87 kg/s，m_L_ (liquid feed rate)=3.47 kg/s (obtained from the material flow data)

Two operating speeds were evaluated: 3000 rpm (314 rad/s) and 8000 rpm (838 rad/s)

Note: The negative battery energy term is treated as a recoverable energy credit under an idealized industrial energy-recovery assumption.

**Table S3** Results of production cost calculation

| Basic Data | | | | | | | | | |
| --- | --- | --- | --- | --- | --- | --- | --- | --- | --- |
| Capacity | | 1000 | kg/h | Shift position | | 3 | | d/y | |
| Plant life | | 20 | y | Numbers of operators per shift | | 5 | | h/y | |
| Annual operating days | | 333 | d/y | Operating labor cost | | 30000 | | $/y | |
| Annual operating hours | | 8000 | h/y | Nominal Interest Rate | | 0.1 | |  | |
| YIELD ESTIMATE | | | | CAPITAL COSTS | | | | | |
| Scale of production set to 1000 kg/h  product unit: kg | | | |  | | | | $ | |
|  |  |  |  | ISBL | | | | 5211333 | |
|  |  |  |  | OSBL | | | | 2084533 | |
|  |  |  |  | Engineering and construction costs | | | | 729587 | |
|  |  |  |  | Contingency charges | | | | 729587 | |
|  |  |  |  | **Fixed Capital Investment (FCI)** | | | | 8755039 | |
| ANNUALIZED CAPITAL CHARGES | | | | | | | | | |
|  | | $ | Annual capital charge ratio(ACCR) | | | $/y | | $/unit product | |
| **Fixed capital investment** | | 8755039 | 0.117 | | | 1028364 | | 0.129 | |
| Total Annual Capital Charge(ACC) | | | | | | 1028364 | | 0.129 | |
| FIXED OPERATING COSTS | | | | | | | | | |
|  | |  |  | | | $/y | | $/unit product | |
| Operating labor | | 15 | Operators: 30000 $/y each | | | 450000 | | 0.056 | |
| Supervision | | 25% | of Operating Labor | | | 112500 | | 0.014 | |
| Direct salary overhead | | 40% | of Labor & Superv | | | 225000 | | 0.028 | |
| Maintenance | | 3% | of ISBL | | | 156340 | | 0.020 | |
| Property taxes and insurance | | 1% | of ISBL | | | 52113 | | 0.007 | |
| General plant overhead | | 65% | of Labor + Superv+Direct salary overhead +Maint. | | | 613496 | | 0.077 | |
| Fixed costs of production (FCOP) | | | | | | 1609449 | | 0.20 | |
| UTILITIES | | | | | | | | | |
|  | Units | Units/Unit product | | Units/y | Price $/units | | $/y | | $/unit product |
| Battery | kwh | -2.80 | | -22400000 | 0.05 | | -1120000 | | -0.14 |
| Ultrasonic bath | kwh | 0.418 | | 3343434 | 0.05 | | 167172 | | 0.0209 |
| Centrifuge | kwh | 0.256 | | 2051370 | 0.05 | | 102568 | | 0.0128 |
| mixer | kwh | 0.0469 | | 375000 | 0.05 | | 18750 | | 0.00234 |
| Total Utilities (UTS) | | | | | | | -831510 | | -0.10 |
| CONSUMABLES | | | | | | | | | |
|  | Units | | | life time(y) | Price $/units | | $/y | | $/unit product |
| PP separator | 5y | | | 5 | 561000 | | 112200 | | 0.0140 |
| Aluminum coater | 5y | | | 5 | 91155 | | 18231 | | 0.0023 |
| Total Consumables (CONS) | | | | | | | 130431 | | 0.016 |
| RAW MATERIAL COSTS | | | | | | | | | |
|  | Units | Units/Unit product | | Units/y | Price $/units | | $/y | | $/unit product |
| TiS_2_ | kg | 2.5 | | 20000000 | 226 | | 4520000000 | | 565 |
| SupperP carbon | kg | 0.3 | | 2500000 | 60 | | 150000000 | | 19 |
| PVDF | kg | 0.3 | | 2500000 | 65 | | 162500000 | | 20 |
| water | kg | 762.5 | | 6100000000 | 0.00054 | | 3294000 | | 0.41 |
| LiFePO4 | kg | 5.5 | | 44000000 | 14 | | 616000000 | | 77 |
| Cu | kg | 1.2 | | 9800000 | 10.5 | | 102900000 | | 13 |
| Al | kg | 0.5 | | 4200000 | 2.1 | | 8820000 | | 1.1 |
| electrolyte | kg | 0.1 | | 1000000 | 305 | | 305000000 | | 38 |
| Total Raw Materials (RM) | | | | | | | 5868514000 | | 734 |
| REVENUES | | | | | | | | | |
| Key Products | Units | Units/Unit product | | Units/y | Price $/units | | $/y | | $/unit product |
| TiS_2_ nanosheets | kg | 1 | | 8000000 | 4300 | | 34400000000 | | 4300 |
| Total Key Product Revenues (REV) | | | | | | | 34400000000 | | 4300 |
| SUMMARY | | | | | | | | | |
|  | | | | | | | $/y | | $/unit product |
| Variable Cost of Production (VCOP = RM + CONS + UTS) | | | | | | | 5867812921 | | 733.5 |
| Fixed Cost of Production (FCOP) | | | | | | | 1609449 | | 0.20 |
| Cash Cost of Production (CCOP) | | | | | | | 5869422371 | | 733.7 |
| Total Annual Capital Charge (ACC) | | | | | | | 1028364 | | 0.13 |
| Gross Profit (GP=REV-CCOP) | | | | | | | 28530577629 | | 3566 |
| Total Cost of Production | | | | | | | 5870450734 | | 733.8 |

**Supplementary References**

[S1] N. Saito, A. Urayama, M. Ikezawa, Y. Kondo, Water-durable cesium lead halide perovskite nanocrystals passivated with a cationic gemini surfactant. Adv. Mater. Interfaces **9**(5), 2101836 (2022). <https://doi.org/10.1002/admi.202101836>
[S2] Y. Deng, Y. Xie, K. Zou, X. Ji, Review on recent advances in nitrogen-doped carbons: preparations and applications in supercapacitors. J. Mater. Chem. A **4**(4), 1144–1173 (2016). <https://doi.org/10.1039/c5ta08620e>
[S3] P. Lu, Y. Xia, G. Sun, D. Wu, S. Wu et al., Realizing long-cycling all-solid-state Li-In||TiS(2) batteries using Li(6+x)M(x)As(1-x)S(5)I (M=Si, Sn) sulfide solid electrolytes. Nat. Commun. **14**(1), 4077 (2023). <https://doi.org/10.1038/s41467-023-39686-w>
[S4] C. Wan, X. Gu, F. Dang, T. Itoh, Y. Wang et al., Flexible n-type thermoelectric materials by organic intercalation of layered transition metal dichalcogenide TiS_2_. Nat. Mater. **14**(6), 622–627 (2015). <https://doi.org/10.1038/nmat4251>
[S5] R. Yang, L. Mei, Q. Zhang, Y. Fan, H.S. Shin et al., High-yield production of mono- or few-layer transition metal dichalcogenide nanosheets by an electrochemical lithium ion intercalation-based exfoliation method. Nat. Protoc. **17**(2), 358–377 (2022). <https://doi.org/10.1038/s41596-021-00643-w>
[S6] M. Ly, T.H. Mekonnen, Cationic surfactant modified cellulose nanocrystals for corrosion protective nanocomposite surface coatings. J. Ind. Eng. Chem. **83**, 409–420 (2020). <https://doi.org/10.1016/j.jiec.2019.12.014>
[S7] V. Vatanpour, S.S. Mousavi Khadem, A. Dehqan, M.A. Al-Naqshabandi, M.R. Ganjali et al., Efficient removal of dyes and proteins by nitrogen-doped porous graphene blended polyethersulfone nanocomposite membranes. Chemosphere **263**, 127892 (2021). <https://doi.org/10.1016/j.chemosphere.2020.127892>
[S8] S. Zhao, Y. Yao, C. Ba, W. Zheng, J. Economy et al., Enhancing the performance of polyethylenimine modified nanofiltration membrane by coating a layer of sulfonated poly(ether ether ketone) for removing sulfamerazine. J. Membr. Sci. **492**, 620–629 (2015). <https://doi.org/10.1016/j.memsci.2015.03.017>
[S9] R. Dai, H. Han, T. Wang, X. Li, Z. Wang, Enhanced removal of hydrophobic endocrine disrupting compounds from wastewater by nanofiltration membranes intercalated with hydrophilic MoS_2_ nanosheets: Role of surface properties and internal nanochannels. J. Membr. Sci. **628**, 119267 (2021). <https://doi.org/10.1016/j.memsci.2021.119267>
[S10] L.-X. Dong, X.-C. Huang, Z. Wang, Z. Yang, X.-M. Wang et al., A thin-film nanocomposite nanofiltration membrane prepared on a support with *in situ* embedded zeolite nanoparticles. Sep. Purif. Technol. **166**, 230–239 (2016). <https://doi.org/10.1016/j.seppur.2016.04.043>
[S11] H. Croll, A. Soroush, M.E. Pillsbury, S. Romero-Vargas Castrillón, Graphene oxide surface modification of polyamide reverse osmosis membranes for improved N-nitrosodimethylamine (NDMA) removal. Sep. Purif. Technol. **210**, 973–980 (2019). <https://doi.org/10.1016/j.seppur.2018.08.070>
[S12] S. Zhao, C. Ba, Y. Yao, W. Zheng, J. Economy et al., Removal of antibiotics using polyethylenimine cross-linked nanofiltration membranes: Relating membrane performance to surface charge characteristics. Chem. Eng. J. **335**, 101–109 (2018). <https://doi.org/10.1016/j.cej.2017.10.140>
[S13] H. Fan, J. Gu, H. Meng, A. Knebel, J. Caro, High-flux membranes based on the covalent organic framework COF-LZU1 for selective dye separation by nanofiltration. Angew. Chem. Int. Ed. **57**(15), 4083–4087 (2018). <https://doi.org/10.1002/anie.201712816>
[S14] Y.T. Nam, J. Choi, K.M. Kang, D.W. Kim, H.-T. Jung, Enhanced stability of laminated graphene oxide membranes for nanofiltration *via* interstitial amide bonding. ACS Appl. Mater. Interfaces **8**(40), 27376–27382 (2016). <https://doi.org/10.1021/acsami.6b09912>
[S15] S.P. Sun, T.A. Hatton, T.-S. Chung, Hyperbranched polyethyleneimine induced cross-linking of polyamide-imide nanofiltration hollow fiber membranes for effective removal of ciprofloxacin. Environ. Sci. Technol. **45**(9), 4003–4009 (2011). <https://doi.org/10.1021/es200345q>
[S16] G.-H. Yang, D.-D. Bao, D.-Q. Zhang, C. Wang, L.-L. Qu et al., Removal of antibiotics from water with an all-carbon 3D nanofiltration membrane. Nanoscale Res. Lett. **13**(1), 146 (2018). <https://doi.org/10.1186/s11671-018-2555-9>
[S17] Q. Dai et al., EverBatt: A closed-loop battery recycling cost and environmental impacts model. Argonne National Laboratory, ANL-19/16 (2019). <https://doi.org/10.2172/1530874>
[S18] G.R.A. Bell, D.D. Symons, J.R. Pearse, Mathematical model for solids transport power in a decanter centrifuge. Chem. Eng. Sci. **107**, 114–122 (2014). <https://doi.org/10.1016/j.ces.2013.12.007>
